# Idiosyncratic patterns of interhemispheric connectivity in the face and scene networks of the human brain

**DOI:** 10.1162/imag_a_00181

**Published:** 2024-05-20

**Authors:** Bartholomew P.A. Quinn, David M. Watson, Kira Noad, Timothy J. Andrews

**Affiliations:** Department of Psychology, University of York, York, United Kingdom

**Keywords:** interhemispheric, intrahemispheric, face, scene, connectivity.

## Abstract

Neuroimaging studies have revealed a network of regions in both hemispheres of the human brain that respond selectively to faces. Neural models of face processing have typically focused on functional connectivity between regions in the same hemisphere (intrahemispheric), with a particular bias toward the right hemisphere. Here, we explored the role of interhemispheric connectivity using fMRI. We used three datasets to compare functional connectivity, as shown by correlations between the time-courses of neural activity of face regions during different natural viewing paradigms. We found higher correlations of neural activity between corresponding interhemispheric regions (e.g., rFFA–lFFA) than between intrahemispheric regions (e.g., rFFA–rOFA), indicating a bias towards higher interhemispheric than intrahemispheric connectivity. A similar interhemispheric bias was evident in scene-selective regions. In contrast, we did not find an interhemispheric bias in early visual regions (V1–V3), where intrahemispheric connectivity between corresponding regions was generally higher than interhemispheric connectivity. Next, we asked whether the higher interhemispheric connectivity in the face and scene networks between corresponding regions was consistent across participants. We found that the interhemispheric bias was significantly attenuated when we compared the time-course of response across participants. This shows that interhemispheric bias in connectivity between corresponding regions in the face and scene networks is specific to the individual. These findings raise the possibility that idiosyncratic variation in interhemispheric connectivity may explain individual differences in perception.

## 1 Introduction

Neuroimaging studies have revealed a number of regions in the human brain which reliably and selectively respond to faces (Kanwisher, 2010). Influenced by cognitive models of face processing (Bruce & Young, 1986,^2012^), neural models propose that the processing of faces occurs primarily through intrahemispheric connections between these face-selective regions (Duchaine & Yovel, 2015;Gobbini & Haxby, 2007;Haxby et al., 2000;Ishai, 2008). The core face network consists of the occipital face area (OFA), the fusiform face area (FFA), and the superior temporal sulcus (STS). A pathway from the OFA to STS is thought to be important for extracting variable aspects of the face used in social interactions (e.g., gaze direction), whereas a pathway from the OFA to FFA is important for extracting invariant facial characteristics used in categorisation and individuation (e.g., identity recognition;Andrews & Ewbank, 2004;Hoffman & Haxby, 2000;Winston et al., 2004). Information from these core regions is then transferred to a network of extended regions for further processing (Gobbini & Haxby, 2007;Haxby et al., 2000). For example, connectivity between the STS and amygdala is thought to be important for the perception of facial expressions of emotion (Harris et al., 2012).

Although face regions are located within both hemispheres, a right-hemisphere bias in face perception is typically reported (Rossion, 2014;Prete & Tommasi, 2018). For example, behavioural studies have shown that faces are better recognised when presented in the left visual field, projecting to the right hemisphere (Bourne & Hole, 2006;Mason & Macrae, 2004;Verosky & Turk-Browne, 2012). Consistent with this behavioural bias, the neural responses to faces across both core and extended face regions are typically stronger in the right hemisphere (Bukowski et al., 2013;Eimer, 2012;Rossion et al., 2012;Yovel et al., 2008). Neuropsychological studies also support a right-hemisphere bias. Unilateral lesions to the right occipital and temporal lobes commonly lead to acquired prosopagnosia (De Renzi et al., 1994;Rossion, 2018), whereas deficits in face recognition following unilateral lesions to the left hemisphere are comparatively rare (Barton, 2008a;Busigny et al., 2010). These findings also fit with studies showing that electrical stimulation of face regions in the right hemisphere leads to a selective disturbance of face perception, whereas corresponding stimulation to the left hemisphere does not (Rangarajan & Parvizi, 2016;Rangarajan et al., 2014).

Nevertheless, a right-hemisphere bias should not be taken to indicate that the left hemisphere does not play a role in face processing. Although prosopagnosia is associated with damage to the right hemisphere, bilateral lesions typically result in greater deficits to face recognition (Barton, 2008b). Moreover, neuroimaging studies have shown reliable left hemisphere responses to faces, even in individuals with a right hemisphere bias (Rossion, 2014;Thome et al., 2022). It has therefore been suggested that the two hemispheres have different but complementary roles in processing faces (Bradshaw & Nettleton, 1981;Rossion & Lochy, 2022;Rossion et al., 2000). For example, the left anterior temporal cortex is involved in accessing verbal and semantic knowledge, whereas the right anterior temporal cortex is thought to be involved in the visual representation and sense of familiarity (Gainotti, 2007).

While the exact functions of the respective hemispheres are debated, the role of communication between hemispheres has been neglected. The importance of interhemispheric connectivity in face perception has been demonstrated by the neurological condition, prosopometamorphopsia, in which the faces appear distorted. This condition typically occurs following damage to white matter tracts connecting face regions across the two hemispheres (Almeida et al., 2020;Blom et al., 2021;Herald et al., 2023). Other support for the importance of interhemispheric connectivity comes from neuroimaging studies. These studies have revealed both functional (Davies-Thompson & Andrews, 2012;Frässle, Krach, et al., 2016;Frässle, Paulus, et al., 2016;Geiger et al., 2016) and structural (Wang et al., 2020) connections between corresponding core regions in the two hemispheres. Despite the absence of interhemispheric connectivity in models of face processing, these studies indicate that connectivity between corresponding regions in opposite hemispheres (e.g., between left FFA and right FFA) often exceeds the magnitude of intrahemispheric connectivity between neighbouring face regions (e.g., between right OFA and right FFA), and may therefore have a critical relevance to the processing of faces.

The aim of this study was to further explore the role of interhemispheric connectivity in face processing. First, we compared interhemispheric connectivity with intrahemispheric connectivity across face regions with fMRI during movie watching. For each dataset, we used a localiser to define bilateral regions of interest (ROIs) within the face network. We correlated within-subject time-courses of BOLD activity between pairs of ROIs to provide a measure of functional connectivity. We compared the magnitude of the functional connectivity between corresponding interhemispheric regions with functional connectivity between intrahemispheric regions. Previous studies investigating functional connectivity of the face network have used experimentally controlled stimulus conditions, such as grey-scaled, static faces presented in blocks (Davies-Thompson & Andrews, 2012;Frässle, Krach, et al., 2016;Frässle, Paulus, et al., 2016;Geiger et al., 2016). However, it is unclear if a similar pattern of response is evident in more naturalistic viewing paradigms (Redcay & Moraczewski, 2020), in which characteristics such as dynamic motion and ambient changes may elicit differentiable neural responses (Kilts et al., 2003;Labar et al., 2003). Here, three movie-watching datasets were used to capture a broader range of dynamic stimuli, providing more ecological validity to participant responses, but also further informing as to if functional connectivity patterns were consistent across different datasets and participant groups. While not the focus of the present study, we also compared the magnitude of interhemispheric connections between non-corresponding regions with intrahemispheric connections to establish if any interhemispheric bias is specific to corresponding regions, as suggested by previous studies (e.g.,Davies-Thompson & Andrews, 2012). Next, we asked whether any established pattern between interhemispheric and interhemispheric connectivity was specific to the face network, by measuring connectivity between higher visual regions in the scene network and between early visual regions (V1–V3). Finally, we asked whether patterns of interhemispheric connectivity were similar across participants or reflected idiosyncratic responses within individuals. To do so, we measured interhemispheric and intrahemispheric functional connectivity between subjects. For both within-subject and between-subject comparisons, the difference between corresponding interhemispheric and highest correlating intrahemispheric correlations for each region was calculated. These were compared to identify if the magnitude of interhemispheric or intrahemispheric bias represented a pattern of activity specific to, or present across, participants.

## 2 Methods

The present study assessed functional connectivity during natural viewing using three existing datasets, each of which involved participants undergoing fMRI during movie-watching, and a task from which face and scene regions could be defined. The “StudyForrest” dataset featured recordings from 15 participants who watched the complete movie “Forest Gump”; in the “Game of Thrones” dataset, 45 participants were recorded while watching 10 short clips from the television series “Game of Thrones”; and in the “Human Connectome Project” dataset, 176 participants watched a series of eight clips from Hollywood movies and eight short independent films.

### 2.1 Studyforrest

#### 2.1.1 Participants

Full participant details can be found in the original dataset publications (Hanke et al., 2014,^2016^;Sengupta et al., 2016), and on the dataset website (http://studyforrest.org/). In this analysis, we included 15 participants who had completed both the audio-visual movie viewing and functional localiser scans. All participants were right-handed (6 female, mean age = 29.4, range 21–39), had normal or corrected-to-normal vision, and spoke German as a native language. All participants provided informed consent, and the study was approved by the Ethics Committee of the Otto-von-Guericke University Magdeburg.

#### 2.1.2 Stimulus

During the movie viewing scan, participants watched the 2-h movie “Forrest Gump” in eight segments, each approximately 15 min in duration, while listening to the official dubbed German audio track (Hanke et al., 2016). They also completed a functional localiser scan in which they viewed greyscale images drawn from six different stimulus categories (human faces, human bodies, objects, houses, outdoor scenes, and phase scrambled images;Sengupta et al., 2016). Stimuli were presented in a block-design. There were 4 runs, each run containing 2 blocks per stimulus category. Each block featured 16 trials presented in random sequence, which were presented for 900 ms, followed by a 100 ms inter-trial-interval. To ensure that attention was maintained on the stimuli, a fixation cross was superimposed throughout the trials and inter-trial-intervals, and participants conducted a one-back task on the images.

#### 2.1.3 fMRI acquisition

Full details on fMRI acquisition and pre-processing can be found in the “StudyForrest” extension publications (Hanke et al., 2016;Sengupta et al., 2016). Both structural and functional scans were acquired using a whole-body 3 Tesla Philips Achieva dStream MRI scanner, with a 32-channel head coil. Structural, movie watching, and localisation scans were all conducted in separate sessions. Structural images (Hanke et al., 2014) were obtained using standard clinical acquisition protocols. T1-weighted images, composed of 274 sagittal slices, were collected via a 3D turbo field echo sequence (TR = 2500 ms, TE = 5.7 ms, FoV = 191.8 x 256 x 256 mm, matrix size = 384 x 384, voxel dimensions = 0.67 x 0.67 mm, slice thickness = 0.7 mm, flip angle = 80°, whole-brain coverage).

For the movie watching (3599 TRs;Hanke et al., 2016) and functional localiser (624 TRs;Sengupta et al., 2016) data, 35 axial slices were collected with a T2*-weighted echo-planar imaging pulse sequence (TR = 2 s, TE = 30 ms, FoV = 240 x 240 mm, matrix size = 80 x 80, voxel dimensions = 3.0 x 3.0 mm, slice thickness = 3.0 mm, flip angle = 90°, whole-brain coverage). Pre-processing was then applied to both movie watching and functional localisation data. This included motion correction using FSL’s MCFLIRT tool (https://fsl.fmrib.ox.ac.uk;Jenkinson et al., 2002), and the alignment of each volume to a common subject-specific reference volume that was shared across all runs.

#### 2.1.4 fMRI analysis

A univariate analysis of the functional localiser data was conducted using FEAT v.6.00 (https://fsl.fmrib.ox.ac.uk). Boxcar regressors were defined for each task condition (faces, headless bodies, objects, houses, scenes, and phase-scrambled images), and convolved with a double-gamma haemodynamic response function. First-level analysis was conducted individually on the four runs from each participant. This included slice timing correction using Fourier-space time-series phase shifting, non-brain removal using BET (Smith, 2002), intensity normalisation, temporal high-pass filtering (σ = 24.0 s), and spatial smoothing (Gaussian) at 6 mm (FWHM). Motion correction parameters provided in the original dataset (Sengupta et al., 2016) were added as explanatory variables. The 4 runs from each participant were concatenated using a higher-level analysis with fixed effects (FLAME,https://fsl.fmrib.ox.ac.uk;Beckmann et al., 2003;Woolrich, 2008;Woolrich et al., 2004). Individual participant data were then entered into a higher-level group analysis using a mixed-effects design in FLAME. Functional images were co-registered to each participant’s T1 anatomical scan via a boundary-based registration algorithm (Greve & Fischl, 2009), and then further to the standard MNI brain (ICBM152) via FSL’s FNIRT tool (Andersson & Skare, 2010).

Movie-watching data were pre-processed in a similar manner to the functional localiser data. First-level analysis was conducted on eight runs of movie-watching data: Slice timing correction using Fourier-space time-series phase shifting, and non-brain removal using BET, grand-mean intensity normalisation of the 4D dataset by a single multiplicative factor, temporal high-pass filtering (σ = 50.0 s), and spatial smoothing (Gaussian) at 6 mm (FWHM) were applied. Higher-level analysis with fixed effects were used to concatenate runs into a single filtered timeseries for each participant, which were normalised by converting to units of percentage signal change and regressing out head motion parameters from the initial dataset (Hanke et al., 2016). Normalised functional data were then transformed from the initial EPI space onto a high-resolution T1-anatomical image drawn from each participant’s individual structural scans (Hanke et al., 2016), before being registered onto the standard MNI brain (ICBM152).

### 2.2 Game of thrones

#### 2.2.1 Participants

Full participant details can be found in the original dataset publication (Noad et al., 2023). We analysed data from 45 neurotypical control participants (30 female, mean age = 19 range 18–32). All participants were right-handed, neurologically healthy, had normal or corrected-to-normal vision, and spoke English as a native language. All participants provided informed consent, and the study was approved by the Ethics Committee of the University of York Neuroimaging Centre.

#### 2.2.2 Stimulus

Participants watched a 778 s compilation of 10 clips taken from the HBO series “Game of Thrones,” while listening to the original English language audio track. Individual clips ranged in length from 50–117 s and were selected to feature a variety of pivotal characters, locations, and scenarios from seasons 3 and 4 of the series. Participants also completed a functional localiser scan. During this scan, participants viewed images drawn from three different stimulus categories (human faces, scenes, and phase-scrambled images). Face stimuli were taken from the Radboud database of face stimuli (Langner et al., 2010), scene stimuli were taken from the SUN database (Xiao et al., 2010), and scrambled images were created by phase-scrambling the face stimuli. Images from each condition were presented in a block design. 12 images were presented in each block, and each image was presented for 600 ms with a 200 ms inter-trials-interval. 9 blocks were presented for each condition in a pseudorandomised order, for a total scan time of 244 s. To ensure that attention was maintained on the stimuli, a fixation cross was superimposed throughout the trials and inter-trial-intervals, and participants were instructed to execute a button-press whenever this cross turned green.

#### 2.2.3 fMRI acquisition

Structural and functional scans were acquired using a whole-body 3 Tesla Siemens MAGNETOM Prisma MRI scanner, with a 64-channel phased array head coil during the same session. MRI structural scans, composed of 176 sagittal slices, were collected via gradient-echo echo-planar imaging pulse sequences (TR = 2300 ms, TE = 2.26 ms, FoV = 240 x 240 mm, matrix size = 256 x 256, voxel dimensions = 1.0 x 1.0 x 1.0 mm, slice thickness = 1.0 mm, flip angle = 60°, whole-brain coverage). Movie watching and functional localiser scans were composed of 60 axial slices collected via T2*-weighted gradient-echo echo-planar imaging pulse sequences (TR = 2 s, TE = 30 ms, FoV = 240 x 240 mm, matrix size = 80 x 80, voxel dimensions = 3.0 x 3.0 x 3.0 mm, slice thickness = 3.0 mm, flip angle = 80°, whole-brain coverage, and phase encoding direction anterior to posterior). Additional field-map images were acquired in the same plane as the functional images (TR = 554 ms, TE = 7.38 ms, flip angle = 60°, other parameters as per functional images).

#### 2.2.4 fMRI analysis

Analysis of the fMRI data was conducted using FEAT. First-level analysis of the movie watching (389 TRs), and functional localiser (122 TRs) data was performed using the following pre-processing steps: motion correction using MCFLIRT, slice timing correction using Fourier-space time-series phase shifting, non-brain removal using BET, temporal high-pass filtering (σ = 50.0 s), and spatial smoothing (Gaussian) at 6 mm (FWHM). Boxcar regressors were defined for each task condition (faces, scenes, and scrambled faces) of the functional localiser scan, and convolved with a double-gamma haemodynamic response function. Individual participant data were then entered into a higher-level group analysis using a mixed-effects design in FLAME. Functional data were co-registered to each participant’s T1 anatomical scan, and then further to the standard MNI brain.

### 2.3 Human connectome project

#### 2.3.1 Participants

Further details on data acquisition and processing can be found in the original dataset publication (Glasser et al., 2016;Van Essen et al., 2013) for the 1200 subjects release for the WU-Minn Human Connectome Project (HCP) dataset (https://www.humanconnectome.org/study/hcp-young-adult/document/1200-subjects-data-release). For the present study, we only analysed data from the 174 participants who had completed 7T movie-watching, resting-state, and retinotopy tasks (seeWatson & Andrews, 2022). All participants (104 female, estimated mean age = 29.39, range 22–36+) were neurologically and physically healthy, and provided informed consent, and the study was approved by the Washington University Institutional Review Board.

#### 2.3.2 Stimulus

Four runs of fMRI data from each participant were recorded over the course of two consecutive days while they watched a series of short movies or movie clips (Cutting et al., 2012). Each run lasted between 14.4 and 15.1 min, as dictated by on the length of the movie stimuli. The stimuli for two of the four runs for each participant were composed of eight (four per run) 1.0–4.0 min-long independent movies licenced under creative commons, dealing with a variety of themes and topics (e.g., “Mrs. Meyer’s Clean Day”—a short documentary focused on urban gardening) with 20 s black-screen intermissions between movies. The stimuli for the remaining two blocks were composed of 3.7–4.3 min-long compilations of six (three per run) Hollywood movies (e.g., Home Alone—3.8 min clip) with 20 s black-screen intermissions between clips. A 1.4 min repeat validation movie played at the end of all runs. On each day of recording, participants watched one run of four independent movies, and one run of three Hollywood movie clips. Functional localisation of face and scene regions was derived from the Human Connectome Project’s Working Memory task, which has previously been shown be an effective means of defining ROIs (Barch et al., 2013;Wang et al., 2020). While undergoing fMRI recording, participants completed two runs of a series of zero-back (responding with a button-press to stimuli matching a target presented at the start of the block), and two-back (responding with a button-press to stimuli matching a target that appeared two trials prior) working memory tasks in response to a series of alternating blocks focused on one of four categories of stimuli (human faces, scenes, tools, and human body parts). Each run consisted of eight category blocks (each category presented twice), and four 15 s fixation blocks. Category blocks were made up of 10 trials, each featuring a 2 s categorical stimulus presentation, followed by 500 ms inter-trial-intervals. The two runs totalled a scan time of 640 s.

#### 2.3.3 fMRI acquisition

Full details on fMRI acquisition and pre-processing can be found in the respective Human Connectome Project publications (Glasser et al., 2013,^2016^;Van Essen et al., 2013). Structural and working memory (localiser) functional scans were acquired using a custom-designed whole-body 3 Tesla Siemens MRI scanner, with a 62-channel phased array receiver coil. Movie-watching functional scans were acquired using a whole-body 7 Tesla Siemens MAGNETOM actively shielded MRI scanner, with a 32-channel receiver head coil array. MRI structural scans, composed of 72 slices, were collected via T1/T2-weighted imaging pulse sequences (TR = 2400/3200 ms, TE = 2.14/565 ms, TI = 1000/0 ms, FoV = 224 x 224 mm, greyordinate spatial resolution = 0.7 mm, image acceleration factor = 2, flip angle = 8°/variable, and whole-brain coverage). Functional localiser scans were composed of 72 slices collected via T2-weighted gradient-echo echo-planar imaging pulse sequences (TR = 810 ms, TE = 33.1 ms, FoV = 208 mm x 180 mm, matrix size = 104 x 90, greyordinate spatial resolution = 2.0 mm, slices = 72, multiband factor = 8, echo spacing = 0.58 ms, and flip angle = 52°). Movie-watching scans were composed of 85 slices collected via T2-weighted gradient-echo echo-planar imaging pulse sequences (TR = 1000 ms, TE = 22.2 ms, FoV = 208 mm x 208 mm, matrix size = 130 x 130, greyordinate spatial resolution = 1.6 mm, slices = 85, multiband factor = 5, image acceleration factor = 2, partial Fourier sampling = 7/8, echo spacing = 0.64 ms, flip angle = 45°, whole-brain coverage, and phase encoding direction alternated between posterior to anterior and anterior to posterior between runs).

#### 2.3.4 fMRI analysis

The present study made use of the minimally pre-processed fMRI data including FIX-ICA denoising provided by the Human Connectome Project (Glasser et al., 2013). As reported by the Human Connectome Project Committee, this pre-processing of the movie watching (3655 TRs) and functional localiser (640 TRs) data was designed to correct for head motion, EPI spatial distortion, and high-pass filtering.

Subcortical data were registered to the MNI brain non-linearly, before using multimodal surface-based alignment (Robinson et al., 2014,^2018^) to register cortical data to the fsLR32k standard surface (Van Essen et al., 2012). Additional spatial smoothing was applied, resulting in FWHM of 3.2 for movie-watching data, and 4 mm for localiser task data. For localiser task data, a high-pass temporal filter (σ = 100.0 s) was additionally applied. Individual participant data were then entered into a higher-level group analysis using the HCP Pipelines (https://www.humanconnectome.org/software/hcp-mr-pipelines/; described inGlasser et al., 2013). Boxcar regressors were defined for each task condition (faces, scenes, bodies, and tools), and convolved with a double-gamma haemodynamic response function. These were entered into a first-level GLM analysis along with temporal derivatives and confound regressors. Using FLAME, parameter estimates were combined over runs for each participant, then subsequently over all participants via higher-level mixed-effects analysis.

### 2.4 Regions of interest

For StudyForrest and Game of Thrones datasets, ROIs were selected and defined based on well-established functional ROIs for faces and scenes. Face regions included the occipital face area (OFA), fusiform face area (FFA), posterior superior temporal sulcus (pSTS), and amygdala (AMG). Scene regions included the occipital place area (OPA), parahippocampal place area (PPA), and retrosplenial cortex (RSC). In the StudyForrest and Game of Thrones analyses, face and scene ROIs were defined using a face>scene or scene>face contrast, respectively. Given the planned between-subject comparisons, to retain the positional consistency and voxel count of the ROIs across participants, within each dataset, ROIs were defined based on functional localiser group averages, rather than for each individual participant. The rationale for using a group localiser was to ensure that the same regions of the brain were analysed for the within- and between-subjects analyses. Seed points for each ROI were defined as the peak voxel within each hemisphere (detailed inSupplementary Section 1.1, StudyForrest; & 1.2, Game of Thrones) at their roughly expected locations indicated by previous research. For a given seed, a flood fill algorithm was used to identify clusters of spatially contiguous voxels around the seed which exceeded a given threshold. The threshold was iteratively adjusted until clusters of approximately 250 voxels (2000 mm^3^) were achieved. This process was repeated for each seed to create masks for each ROI. Although previous studies have found that the FFA can be divided into subdomains (Çukur et al., 2013;Weiner & Zilles, 2016), it was not possible to effectively define 250 voxel clusters isolated to subdomains from the group analysis and clustering methodology.

The Human Connectome Project’s greyordinate-based space uses cortical surface vertices. To generate comparably sized ROIs to our other studies, we initially projected the 250 voxel clusters used in other experiments to the FreeSurfer fsaverage brain. From this, we calculated an average ROI surface area of 382.42 mm^2^, rounded to 380 mm^2^as the expected size of our Human Connectome Project ROIs. Again, face and scene ROIs were defined using a “face>scene” and “scene>face” contrast respectively, drawn from the working memory task. A seed vertex was used to identify clusters of spatially contiguous surface vertices which exceeded a given threshold, this time iteratively adjusted until surface clusters of 380 mm^2^were achieved. This process was repeated for each seed to create masks for each ROI, with the exception of the subcortical amygdala, which was generated as a 250 voxel (2000 mm^3^) cluster in the same manner as described above (detailed inSupplementary Section 1.3for details).

In addition to face and scene ROIs, we also defined ROI masks for early visual areas within the dorsal (d) and ventral (v) pathways (V1d, V2d, V3d, V1v, V2v, V3v) based upon visual field masks generated byWang et al. (2015). For StudyForrest and Game of Thrones datasets, 250 voxel clusters were created within these masks using the full probability maps provided byWang et al. (2015). Seed points for each ROI were defined as the peak probability of being a given early visual area, and a flood fill algorithm was used to identify clusters of high-probability spatially contiguous voxels around the seed, restricted within their respective Wang et al. masks. For the Human Connectome Project Dataset, early visual areas were also defined based on the full dimension Wang et al. visual field masks using the Benson Neuropythy pipeline (https://nben.net/software), further details of which are described inBenson and Winawer (2018).

The locations of ROIs used in the connectivity analysis are shown inFigure 1. Further details of the regions are provided inSupplementary Section 1.

**Fig. 1.**
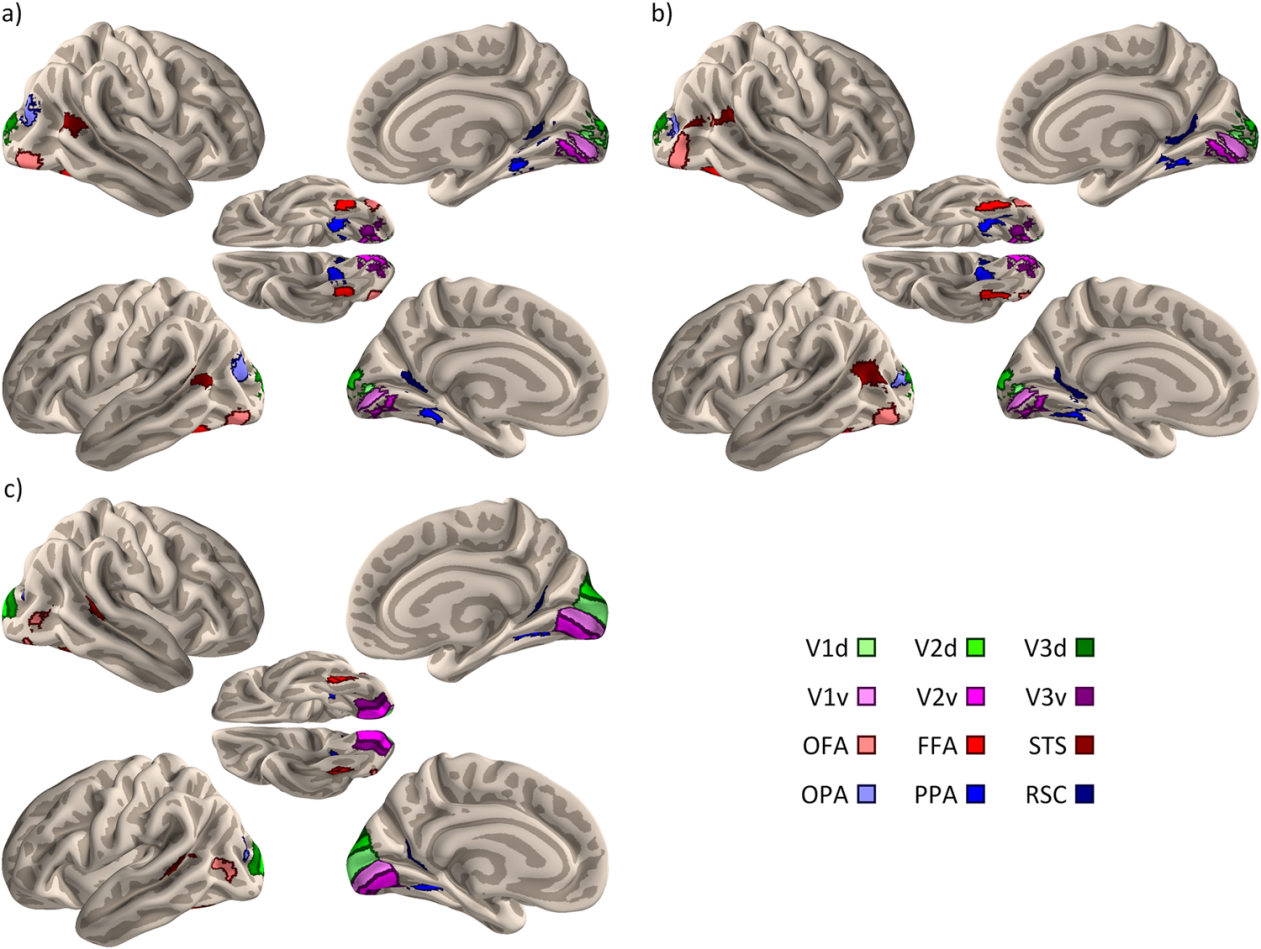
FreeSurfer surface projections of regions of interest defined using group-averaged localisers specific to each dataset within the early visual dorsal (V1d; V2d; V3d), early visual ventral (V1v; V2v; V3v), face (OFA, occipital face area; FFA, fusiform face area; STS, superior temporal sulcus) and scene (OPA, occipital place area; PPA, parahippocampal place area; RSC, retrosplenial cortex) networks. Early visual regions were based on visual field masks (Wang et al., 2015). Face and scene regions were generated from separate localiser scans for the (a) Studyforrest, (b) Game of Thrones datasets, and (c) the Human Connectome Project dataset.

### 2.5 Functional connectivity analysis

For all datasets, time-courses of activity were normalised by converting to units of percent signal change, and for the StudyForrest and HCP datasets were additionally concatenated over scan runs. The normalised time-courses of activity during movie watching were averaged over voxels within each ROI. For each examined ROI within a given network, this averaged time-course of activity was correlated with the activity of other ROIs within the same functional network, the strength of correlation being taken as a measure of functional connectivity (seeFriston et al., 1997;Hampson et al., 2004;Hlinka et al., 2011).

As illustrated inFigure 2, we compared interhemispheric connectivity revealed by correlations between corresponding interhemispheric regions (e.g., lOFA:rOFA) with intrahemispheric connectivity revealed by (1) the averaged intrahemispheric correlations between regions within the face network, and (2) the highest correlating intrahemispheric pairing with this region (e.g., rOFA:rFFA). While not the main focus of the present study, correlations between non-corresponding interhemispheric regions (e.g., lOFA:rFFA) were also calculated, and their network averaged and highest correlating pairs were also compared with their intrahemispheric counterparts. Pearson’s*r*correlation values were converted into Fisher’s*Z*values (*Zr*) prior to statistical analysis, and the magnitude of participant correlations was compared using paired-sample*t-*tests. To examine if similar patterns of bias existed for regions within other networks, the same analyses were conducted for regions in the scene-selective and early visual networks. To account for multiple comparison corrections, Holm-Bonferroni corrections that were applied were adjusted for the number of regions within each network * 2 intrahemispheric (vs. average, and vs. highest correlating pair) correlation types (face network: 8 corrections, scene network: 6 corrections, early visual network: 12 corrections).

**Fig. 2.**
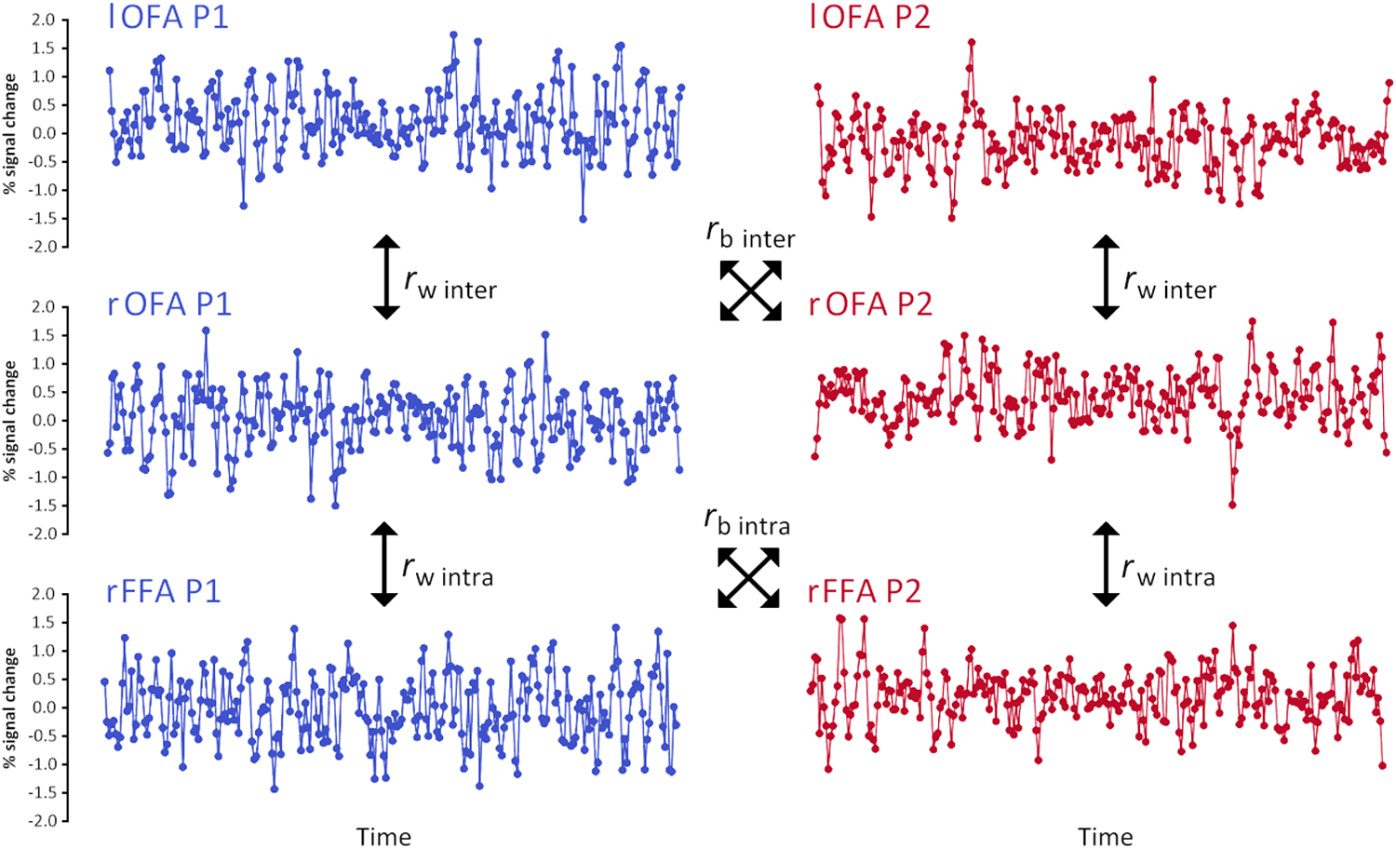
Interhemispheric (_inter_) and intrahemispheric (_intra_) time-course of activity correlations from two exemplar brain regions (OFA – Occipital Face Area; FFA – Fusiform Face Area). These correlations were performed for all regions either within individual participants (r_w_) or between different participants (r_b_). Within-subject correlations were averaged across all participants. Between-subject correlations were averaged across all possible combinations of all participants. These analyses were repeated for all regions within the early visual, face, and scene networks.

To determine whether the interhemispheric bias was specific to individual participants, the same analysis was performed between participants. Here, for each participant, regional activation was compared with the activation from other participants in the dataset, based on the given connectivity comparison. For example, for corresponding interhemispheric connectivity of the OFA, the activity of P1’s lOFA was correlated with the activity of all other participants’ rOFA, and repeated for the reverse (P1 rOFA:others lOFA). These were then averaged into a single correlation for a given region and participant. This process was repeated for all participants, regions, and comparisons (interhemispheric corresponding, interhemispheric non-corresponding, intrahemispheric average, and intrahemispheric highest correlating pairing) for each dataset, and corrections for multiple comparisons were applied as described above. The difference between interhemispheric and intrahemispheric connectivity for each region both within- and between-subjects was calculated and compared using independent*t*-tests to indicate if any biases identified in within-subjects comparisons represented an idiosyncratic or more general pattern of functional connectivity.

## 3 Results

### 3.1 Interhemispheric and intrahemispheric connectivity in the face network

To assess functional connectivity, we first compared the magnitude of interhemispheric and intrahemispheric correlations between face regions during 3 natural viewing paradigms (StudyForrest, Game of Thrones, & Human Connectome Project). We focused on interhemispheric connectivity between corresponding regions (e.g., rOFA:lOFA). Instances in which interhemispheric connectivity has been calculated between non-corresponding regions (e.g., rOFA:lFFA) is referred to with the “non-corresponding” prefix. Results of these analyses are illustrated inFigure 3and reported in full inSupplementary Sections 2.1, 4.1, and 6.

**Fig. 3.**
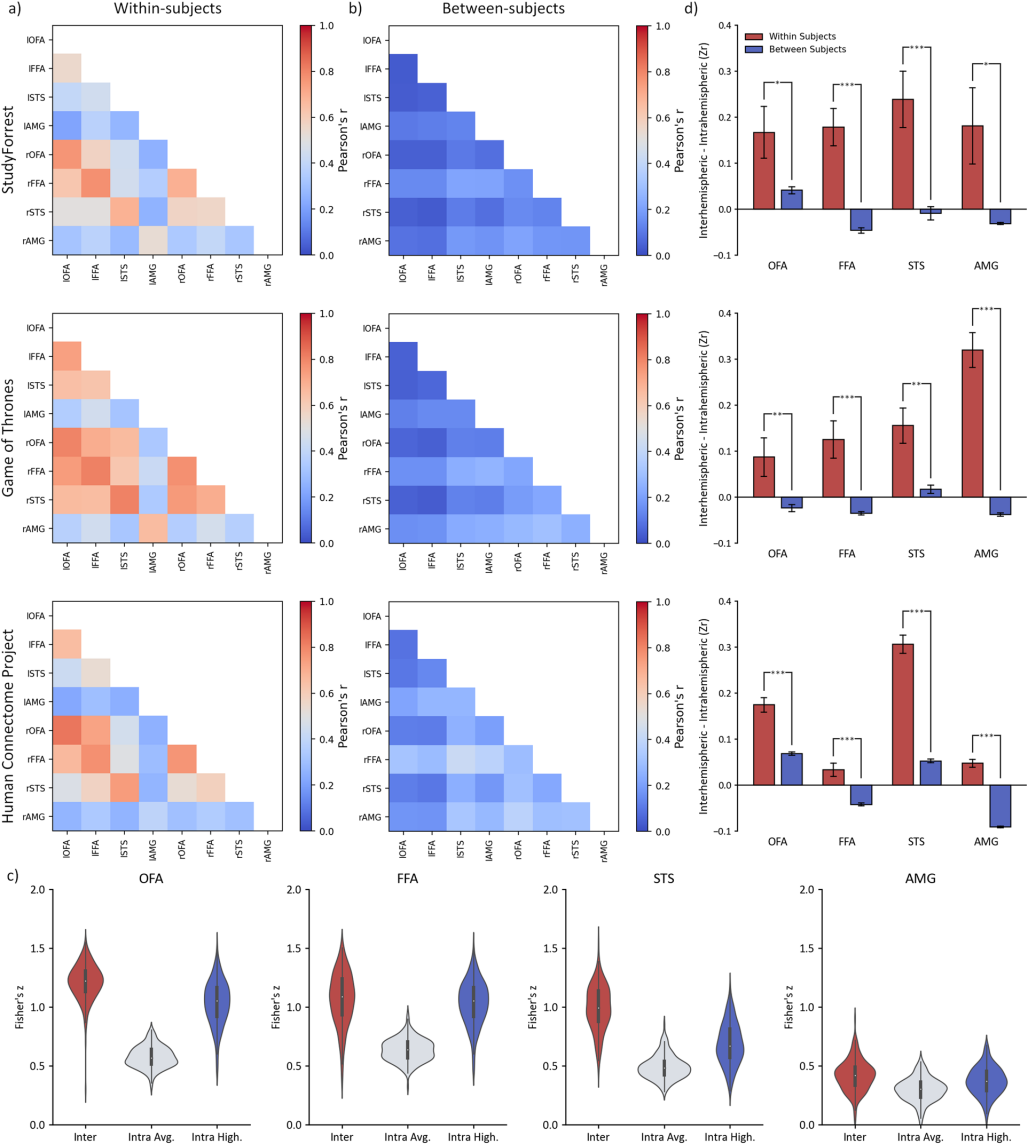
Interhemispheric functional connectivity between corresponding regions and intrahemispheric functional connectivity across face regions for StudyForrest, Game of Thrones, and Human Connectome Project datasets. Data were analysed (a) within-subjects or (b) between subjects. (c) violin plot showing participant average and distribution of data used in*t*-test comparisons: interhemispheric corresponding, intrahemispheric average, and intrahemispheric highest correlations for face regions visualised here for the Human Connectome Project dataset (d) Significantly higher interhemispheric correlations were evident between corresponding regions (e.g., lOFA:rOFA) compared to the highest intrahemispheric (e.g., lOFA:lFFA) correlations in the within-subject analysis. However, this was attenuated or not evident in the between-subjects analysis. This suggests the idiosyncratic nature of interhemispheric connectivity for regions within the face network. Error bars reflect standard error.

#### 3.1.1 Within-subjects connectivity

Our first aim was to compare the magnitude of interhemispheric and intrahemispheric connectivity within each subject (Fig. 3a, c). Within the face network, we found significantly higher interhemispheric correlations compared to averaged intrahemispheric correlations for all regions across all datasets (*p*< .001). To address the possibility that averaging may have masked individual intrahemispheric correlations, we also compared interhemispheric correlations with the highest correlating intrahemispheric pairings for each region.Supplementary Section 2.1shows that interhemispheric correlations were of significantly greater magnitude for all regions when compared to the highest correlating intrahemispheric regions across all datasets (*p *≤ .046).

#### 3.1.2 Between-subjects connectivity

Next, we measured the magnitude of interhemispheric and intrahemispheric connectivity between participants (Fig. 3b). Here, for each ROI, the time course of activity for each participant was correlated with time courses of all other participants and averaged to create single between-subject region pairing correlations.Supplementary Section 4.1shows the paired sample*t*-tests that compared correlations between corresponding left and right hemisphere ROIs. Although we found consistently higher interhemispheric compared to averaged intrahemispheric correlations (*p*< .001) for all face regions except the amygdala, the magnitude of this effect was much smaller when compared to the within-subjects analysis. When interhemispheric correlations were compared with correlations between the highest-correlated intrahemispheric region pairings, a far less consistent pattern emerged, with the FFA and AMG showing consistently higher intrahemispheric connectivity (*p*< .001), but an inconsistent direction and significance across the OFA and STS across datasets (e.g., OFA Studyforrest - [*t*(14) = 5.46,*p*< .001,*d_avg_*= 1.06]; OFA Game of Thrones [*t*(44) = -3.03,*p*= .008,*d_avg_*= 0.37]).

#### 3.1.3 Comparison of within-subjects and between-subjects connectivity

Although face regions showed higher interhemispheric correlations than any intrahemispheric pairing within-subjects, this was less consistent in the between-subjects. To measure this directly, we compared within-subjects and between-subjects correlations by performing a paired-sample*t-*test on the difference between*Interhemispheric*(Zr)–*Intrahemispheric High*(Zr) correlations for each subject. Results of this analysis are visualised inFigure 3cand reported in full inSupplementary Section 6. Across all face-sensitive regions, for StudyForrest (*p*< .046), Game of Thrones (*p*< .008), and Human Connectome Project (*p*< .001) datasets, the correlation differences between interhemispheric and intrahemispheric regions were significantly greater for within-subject than between-subject comparisons.

### 3.2 Interhemispheric and intrahemispheric connectivity in the scene network

To further understand if the established pattern of interhemispheric connectivity extended to the scene-selective network, we repeated the previously described analyses on scene selective regions. Results of these analyses are illustrated inFigure 4and reported in full inSupplementary Sections 2.2, 4.2, and 6.

**Fig. 4.**
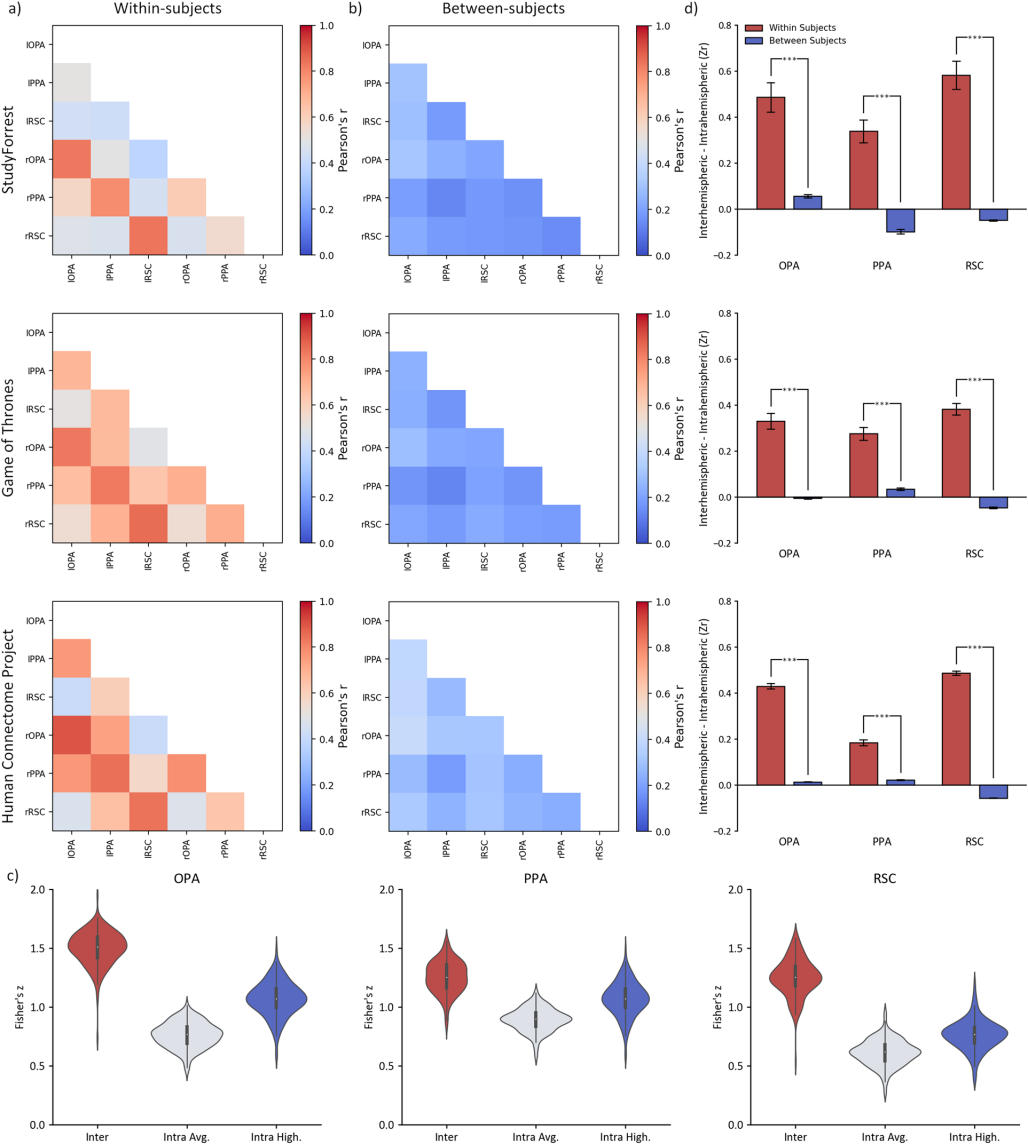
Interhemispheric functional connectivity between corresponding regions and intrahemispheric functional connectivity across scene regions for StudyForrest, Game of Thrones, and Human Connectome Project datasets analysed (a) within-subjects or (b) Between-subjects comparisons. (c) violin plot showing participant average and distribution of data used in*t*-test comparisons: interhemispheric corresponding, intrahemispheric average, and intrahemispheric highest correlations for scene regions visualised here for the Human Connectome Project dataset (d) Significantly higher interhemispheric correlations were evident between corresponding regions (e.g., lOPA:rOPA) compared to the highest intrahemispheric (e.g., lOPA:lPPA) correlations in the within-subject analysis. However, this was attenuated or not evident in the between-subjects analysis. This suggests the idiosyncratic nature of interhemispheric connectivity for regions within the scene network. Error bars reflect standard error.

#### 3.2.1 Within-subject connectivity

We compared the interhemispheric correlations with the averaged and highest correlating intrahemispheric pairings within each subject (Fig. 4a, c). Across all three datasets, interhemispheric correlations for regions within the scene network were significantly greater than the averaged and highest correlating intrahemispheric regions (*p*< .001;Supplementary Section 2.2).

#### 3.2.2 Between-subjects connectivity

We also measured the magnitude of corresponding interhemispheric and intrahemispheric correlations between participants (Supplementary Section 4.2). Although both OPA and PPA showed higher interhemispheric correlations compared to the averaged intrahemispheric correlations across all three datasets (*p*≤ .010), this difference was smaller than in the within-subjects analysis. The RSC showed an inconsistent pattern, with higher averaged intrahemispheric correlations in the StudyForrest dataset [*t*(14) = -4.01,*p*= .002,*d_avg_*= 0.40]; higher averaged interhemispheric correlations in the Game of Thrones dataset [*t*(44) = 3.97,*p*< .001,*d_avg_*= 0.24]; and no significant difference in the Human Connectome Project dataset [*t*(173) = -0.08,*p*= .937,*d_avg_*= 0.00].

Corresponding interhemispheric correlations were also compared with the highest-correlating intrahemispheric region pairings. The RSC showed consistently higher intrahemispheric compared to highest interhemispheric correlations (*p*< .001). Contrastingly, the OPA showed higher interhemispheric correlations, but these were only significant in the StudyForrest and Human Connectome Project datasets (both*p*< .001). The difference between the interhemispheric and the highest intrahemispheric correlations in the PPA was inconsistent across datasets, showing significant differences in both directions (e.g., StudyForrest PPA [*t*(14) = -10.27,*p*< .001,*d_avg_*= 2.18]; Game of Thrones PPA [*t*(44) = 6.97,*p*< .001,*d_avg_*= 0.57].

#### 3.2.3 Comparison of within-subjects and between-subjects connectivity

Next, we compared the within-subjects and between-subjects analysis (seeFig. 4c&Supplementary Section 6). Comparisons were again performed on the difference between the interhemispheric and highest intrahemispheric correlations from the within-subjects and between-subjects analysis. Across all scene-sensitive regions for all datasets, the within-subject differences were significantly greater than the between-subject differences (all*p*< .001).

### 3.3 Interhemispheric and intrahemispheric connectivity in the early visual network

Finally, we compared interhemispheric and intrahemispheric correlations within early visual regions. Results of these analyses are illustrated inFigure 5and reported in full inSupplementary Sections 2.3, 4.3, and 6.

**Fig. 5.**
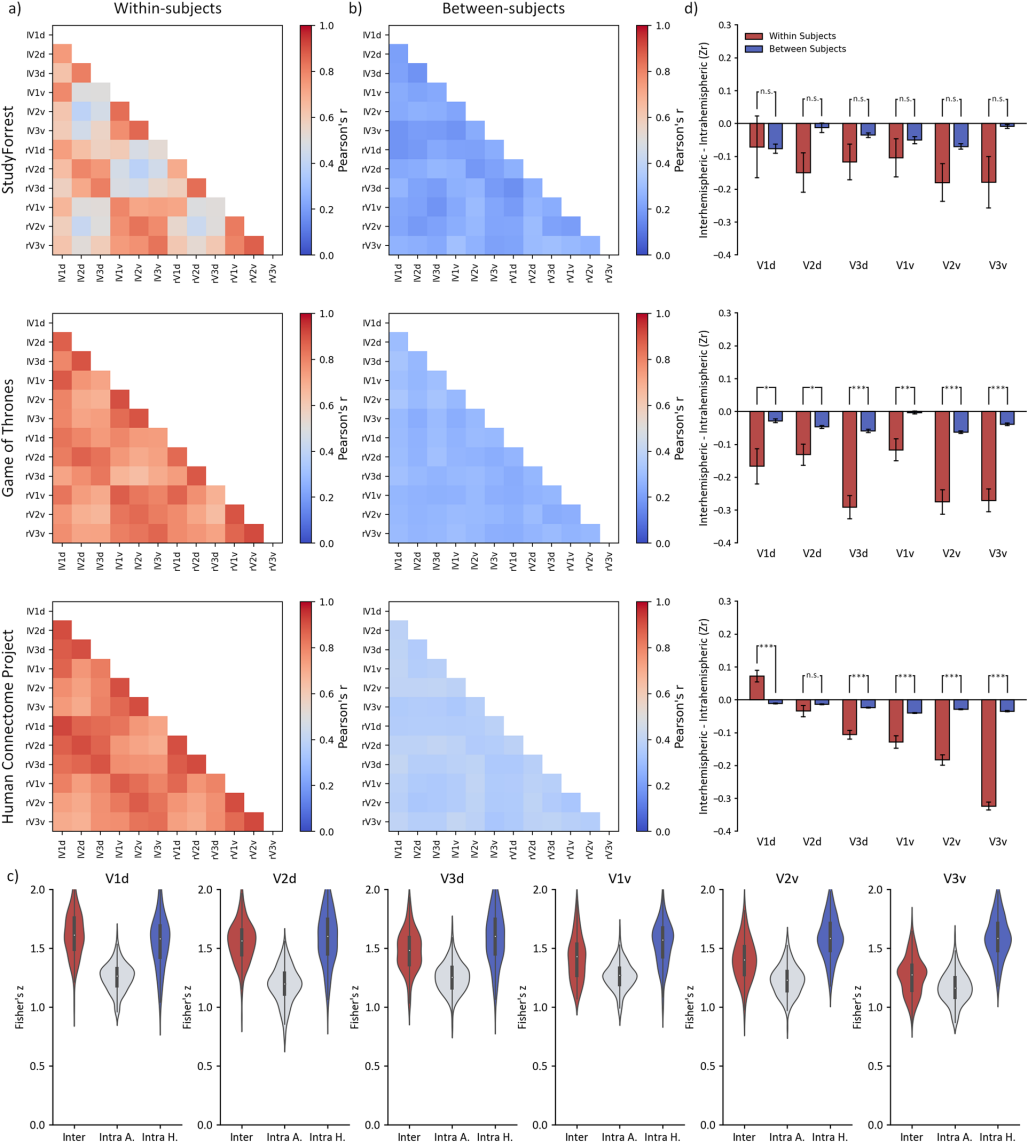
Interhemispheric functional connectivity between corresponding regions and intrahemispheric functional connectivity across early visual regions for StudyForrest, Game of Thrones, and Human Connectome Project datasets analysed (a) within-subjects or (b) Between-subjects comparisons. (c) violin plot showing participant average and distribution of data used in*t*-test comparisons: interhemispheric corresponding, intrahemispheric average, and intrahemispheric highest correlations for early visual regions visualised here for the Human Connectome Project dataset (d) In contrast to the face and scene regions, the highest intrahemispheric (e.g., lV1d:lV2d) correlations were generally higher than interhemispheric correlations between corresponding regions (e.g., lV1d:rV1d) correlations. Error bars reflect standard error.

#### 3.3.1 Within-subject connectivity

First, we compared interhemispheric correlations with the average and highest intrahemispheric correlations within each individual subject (Fig. 5a, c). Save for the V1d in the StudyForrest [*t*(14) = 2.65,*p*= .114,*d_avg_*= 0.68]; and Game of Thrones [*t*(44) = 1.96,*p*= .057,*d_avg_*= 0.29] datasets, across all three datasets, interhemispheric correlations for early visual regions were greater than averaged intrahemispheric correlations (*p*≤ .008). However, when interhemispheric correlations were compared with the highest correlating intrahemispheric pairings, intrahemispheric connectivity was higher across all except one region (Human Connectome Project V1d [*t*(173) = 4.08,*p*< .001,*d_avg_*= 0.35]) in the Game of Thrones and Human Connectome project datasets (*p*< .049). StudyForrest correlations also trended in this direction, but did not reach significance (*p*≥ 0.56;Supplementary Section 2.3).

#### 3.3.2 Between-subjects connectivity

We also measured the magnitude of corresponding interhemispheric and intrahemispheric correlations between participants (Fig. 5b). All regions except the StudyForrest V1d [*t*(14) = 2.02,*p*= .189,*d_avg_*= 0.23] showed significantly higher magnitude interhemispheric correlations when compared to averaged intrahemispheric correlations (*p*≤ .024). However, when compared to the highest intrahemispheric pairings within the early visual network, intrahemispheric correlations were predominantly of greater magnitude than interhemispheric correlations (*p*≤ .003), only failing to reach significance in this direction for the StudyForrest dataset V2d, V3v, and Game of Thrones dataset V1v (*p*≥ .252;Supplementary Section 4.3).

#### 3.3.3 Comparison of within-subjects and between-subjects connectivity

In contrast to the face and scene networks, regions within the early visual networks demonstrated interhemispheric correlations of lower or comparable magnitude when compared to the highest correlating intrahemispheric pairings. We compared the difference between interhemispheric and intrahemispheric connectivity in the within-subjects and between-subjects analysis. Results are visualised inFigure 5cand reported inSupplementary Section 6. For the Game of Thrones and Human Connectome Project datasets, higher intrahemispheric compared to interhemispheric correlations were evident in the within-subjects analysis across all regions (*p*< .018) except the Human Connectome Project V1d [*t*(173) = 4.76,*p*< .001,*d_avg_*= 0.50]; and V2d [*t*(173) = -1.19,*p*= .235,*d_avg_*= 0.13]. However, no differences reached significance across the StudyForrest dataset (*p*> .174).

### 3.4 Connectivity of non-corresponding interhemispheric regions

While the present study focused on interhemispheric functional connectivity between corresponding regions, interhemispheric correlations between non-corresponding regions (e.g., lOFA:rFFA; lOPA:rPPA; lV1d:rV2d) were also calculated and compared with intrahemispheric correlations for all networks, datasets, and subject comparisons. In contrast to the interhemispheric bias between corresponding regions, we did not find a similar interhemispheric bias between non-corresponding regions. While not the focus of our present study, these data are reported inSupplementary Sections 3.1 and 5.1(Face Network);3.2 and 5.2(Scene Network); and3.3 and 5.3(Early Visual Network).

## 4 Discussion

The aim of this study was to investigate the importance of interhemispheric connectivity in face processing during natural viewing. The main findings from this study are: (1) interhemispheric connectivity between corresponding face regions (e.g., rFFA:lFFA) was greater than intrahemispheric connectivity (e.g., rOFA:rFFA); (2) a similar interhemispheric bias was evident in the scene processing network, but was not evident in early visual regions (V1–V3); (3) interhemispheric bias of the face and scene regions was significantly attenuated in a between-subjects analysis, implying that it reflects idiosyncratic neural responses.

Models of face perception focus on the importance of connectivity between regions in the same hemisphere (Duchaine & Yovel, 2015;Haxby et al., 2000;Ishai, 2008). This focus has been encouraged by a range of evidence reporting a right hemisphere dominance for face processing (Rossion, 2014;Prete & Tommasi, 2018), although other studies indicate that the role of the left hemisphere in the perception of faces is not negligible (Barton, 2008b;Bradshaw & Nettleton, 1981;Thome et al., 2022). Despite the focus on intrahemispheric connectivity, interhemispheric connectivity may also play a critical role in face processing. For example, several studies have shown strong interhemispheric connectivity between corresponding face-selective regions in response to static face stimuli (Davies-Thompson & Andrews, 2012;Frässle, Krach, et al., 2016;Frässle, Paulus, et al., 2016;Geiger et al., 2016). We extend these findings by showing strong interhemispheric covariation in neural responses between corresponding regions of the face network that is consistent across different natural viewing paradigms, with all regions across all datasets showing significantly greater correlations of neural response between corresponding interhemispheric regions than even the highest correlating intrahemispheric regional pairings. Given that regions showing greater correlations in activity are more functionally connected (Friston et al., 1997;Hampson et al., 2004,Hlinka et al., 2011), our findings indicate that there are strong functional connections between corresponding regions in the two hemispheres, consistent across a range of dynamic viewing experiences representative of naturalistic viewing.

A key finding from our study is that interhemispheric connectivity between corresponding regions is stronger than intrahemispheric connectivity, even if the comparison is made with the highest intrahemispheric within-network connection. It is important to note that this high interhemispheric connectivity was only evident between corresponding regions, such as the left and right FFA. While not the primary focus of the present study, interhemispheric correlations between non-corresponding regions, such as the left FFA to the right OFA, were also calculated and compared to interhemispheric correlations. These non-corresponding interhemispheric connections did not exhibit the same interhemispheric bias as corresponding regions (seeSupplementary Section 3), showing overall weaker connectivity than intrahemispheric pairings for both averaged and highest correlating region pairs.

The strong interhemispheric connectivity between corresponding regions (hereon abbreviated to “interhemispheric connectivity”) that we show in this study may play an important role in binding face halves into a unified perceptual representation. When viewing faces, we typically fixate on the horizontal mid-line of the face (Peterson & Eckstein, 2012;Walker-Smith et al., 2013). Despite some overlap, this means that the left half of a face is predominantly processed by the right hemisphere, and the right half of the face is predominantly processed by the left hemisphere (Hsiao & Cottrell, 2008). Face-selective regions exist within both hemispheres, and regions in the core face network have been shown to preferentially process information from the contralateral visual field (Silson, Groen, et al., 2016;Silson et al., 2022). Given these lateralised representations of faces, interhemispheric connectivity may play an important role in integrating information about faces into a holistic representation (Richler et al., 2014;Rossion, 2013;Tanaka & Farah, 2006). The importance of integrating information across the hemispheres is apparent in lesions to the splenium of the corpus callosum that can result in a condition known as prosopometamorphopsia, in which faces are perceived as distorted (Almeida et al., 2020;Blom et al., 2021;Herald et al., 2023). While our methodology did not allow us to draw conclusions regarding finer grained subdomains of face-selective regions (e.g., the FFA:Çukur et al., 2013;Weiner & Zilles, 2016), a recent study showed that subdivisions of the FFA display differences in face specificity and intra-hemispheric functional connectivity (Chen et al., 2023). As such, a promising direction for future research could be to explore interhemispheric connectivity in these sub-divisions with greater specificity. Nonetheless, the observed patterns suggest that interhemispheric communication is an important aspect of processing in the core perceptual regions of the face network. Our finding of strong interhemispheric connectivity between the right and left amygdala suggests that interhemispheric connectivity may not be limited to perceptual integration, but also has relevance to the associative roles of the extended network.

Our results also show that stronger interhemispheric compared to intrahemispheric connectivity in the face network was specific to individuals. For example, although we found high correlations of activity between the left and right FFA within individual subjects, this was significantly attenuated when we compared the left FFA and right FFA from different individuals. Moreover, this reduction was much greater for interhemispheric compared to intrahemispheric connectivity. This suggests that the interhemispheric connectivity that we observe is more idiosyncratic than intrahemispheric connectivity. Significant inter-individual differences in face perception are evident across different face tasks (Hamann & Canli, 2004;White & Burton, 2022), and previous studies have identified a relationship between these individual differences in ability and connectivity between face-selective regions. For example,Levakov, Sporns, and Avidan (2022)identified positive correlations between individual differences in the magnitude of structural and functional connectivity of the face network and face recognition ability. Reduced structural and functional connectivity in the occipito-temporal cortex (Rosenthal et al., 2017;Thomas et al., 2009) has been identified as a potential marker of prosopagnosia, with increasing hyper-connectivity in more posterior regions of the visual cortex correlating with the severity of recognition deficit (Rosenthal et al., 2017). However, only a few studies to date have compared the relationship between functional connectivity and individual differences in ability, and none of these have focused on interhemispheric connectivity. Although we are unable to address this question from the results of the analyses performed in the current study, it is interesting to speculate whether the idiosyncratic variance in interhemispheric connectivity that we show might underpin individual differences in behaviour.

The present study defined regions of interest using group averages of neural activity within each dataset. This allowed us to maintain homogeneity in position and size of these regions, of particular relevance to our between-subject comparisons. However, individual differences in higher-level perceptual regions’ sizes and positions have been previously demonstrated (e.g.,Glezer & Riesenhuber, 2013), which are likely to have been overlooked by our methodology. A cursory analysis demonstrated that our group-average within-subject findings were consistent with a small sample of individually defined regions of interest. However, we suggest that examining the variability of connectivity for individually defined regions is an important direction for future research, particularly in identifying a link between idiosyncratic interhemispheric connectivity and potential behavioural correlates.

Higher interhemispheric compared to intrahemispheric connectivity was also evident in the scene network, with all regions across all datasets showing a consistent pattern of significant differences in the within-subjects comparison. Neuroimaging studies have revealed a network of regions that are selective for scenes (Dilks et al., 2022;Epstein & Baker, 2019), which when damaged commonly leads to impairments in scene perception and spatial navigation (Aguirre & D’Esposito, 1999;Mendez & Cherrier, 2003). These regions include the parahippocampal place area (PPA;Epstein & Kanwisher, 1998); the retrosplenial cortex (RSC), located superior to the PPA (Maguire, 2001); and the occipital place area (OPA) on the lateral occipital lobe (Dilks et al., 2013). Functional connectivity studies of the scene network suggest that it can be divided into an anterior and posterior subdivisions (Baldassano et al., 2016,Nasr et al., 2013;Silson, Steel, et al., 2016). Similar to the face network, responses in scene-selective regions are stronger to stimuli presented in the contralateral versus ipsilateral hemifield (Groen et al., 2017;MacEvoy & Epstein, 2007). One role of interhemispheric connectivity may be to bind the representations of scenes across the two hemispheres. While the present study limited analysis to only higher-level visual regions selective to faces and scenes, the possibility of a similar role of hemifield binding for both networks may suggest that a high level of interhemispheric connectivity is a more general property of higher-level visual processing.

The bias toward interhemispheric connectivity was not evident in early visual areas (V1–V3). In these regions, intrahemispheric connectivity was higher than interhemispheric connectivity in all regions across the Game of Thrones and Human Connectome Project datasets. Higher intrahemispheric correlations were also indicated in the StudyForrest dataset, but these were not significant. However, this may have been due to the dataset’s lower participant count (*n*= 15). These results fit with studies from monkeys showing that V1 and V2 in each hemisphere are only interconnected across their representation of the vertical meridian (Hof et al., 1997;Hubel & Wiesel, 1967;Kennedy et al., 1986). Evidence from lesion studies (Holmes, 1945), retinotopic mapping (Engel et al., 1997), and population receptive field methods (Dumoulin & Wandell, 2008) indicates highly lateralised responses in these early visual regions. However, unlike higher-level visual regions, there is little indication of other functional differences between the hemispheres at this stage of processing.

The aim of this study was to measure patterns of functional connectivity during naturalistic viewing. This contrasts with the approach used in other studies which have measured stimulus-free functional connectivity during rest (seevan den Heuvel & Hulshoff Pol, 2010). Despite the more cognitively demanding nature of natural viewing tasks, these have still been shown to elicit synchronised neural responses across participants (Hasson et al., 2004,^2010^) which may be more reliable and behaviourally predictive than those generated in resting-state (Finn & Bandettini, 2021;Wang et al., 2017). Nonetheless, results from studies examining functional connectivity during resting state have also shown strong interhemispheric functional connectivity between corresponding regions in the face network (Geiger et al., 2016).

While also not examined in the present study, our findings predict strong interhemispheric white matter connections between corresponding regions in the face network. AlthoughWang et al. (2020)identified structural connections between corresponding interhemispheric regions in the face network, the interhemispheric fibre counts were largely outweighed by those of intrahemispheric connections. This disparity may reflect limitations in the ability of diffusion tractography to measure longer-distance pathways between hemispheres, and further research is needed to consolidate interhemispheric functional with structural connectivity.

In conclusion, we found consistent evidence across different natural viewing paradigms for higher interhemispheric compared to intrahemispheric connectivity in both the face and scene networks. A similar pattern was not evident in early visual areas. Although we did not investigate other networks in the visual brain, we predict a similar interhemispheric bias. This interhemispheric bias was most evident when we compared responses within individuals. Future research might seek to determine whether the idiosyncratic nature of this interhemispheric bias reflects individual differences in tasks of face and scene processing.

## Supplementary Material

Supplementary Material

## Data Availability

All analysis code is available on the OSF (https://osf.io/w6dmx/). All sets of MRI data were obtained from already publicly available repositories: StudyForrest project dataset (https://www.studyforrest.org/) Game of Thrones dataset (https://openneuro.org/datasets/ds004848) Human Connectome Project dataset (https://db.humanconnectome.org/)
