# Supplementary material for: Idiosyncratic patterns of interhemispheric connectivity in the face and scene networks of the human brain
Source: Imaging Neurosci (Camb). 2024 May 20;2:imag-2-00181. doi: 10.1162/imag_a_00181 (PMC12247604; doi:10.1162/imag_a_00181)
Supplement: Supplementary Material [file imag_a_00181-supp.pdf]

SUPPLEMENTARY MATERIAL

Contents

METHODS 2
1. Regions of Interest 2
1.1 StudyForrest dataset 2
1.2 Game of Thrones dataset 4
1.3 Human Connectome Project dataset 5
RESULTS 7
2. Within-subject interhemispheric corresponding vs. intrahemispheric 7
2.1 Face Network 7
2.2 Scene Network 11
2.3 Early Visual Network 12
3. Within-subject interhemispheric non-corresponding vs. intrahemispheric 14
3.1 Face Network 16
3.2 Scene Network 18
3.3 Early Visual Network 20
4. Between-subject interhemispheric corresponding vs. intrahemispheric 22
4.1 Face Network 24
4.2 Scene Network 26
4.3 Early Visual Network 28
5. Between-subject interhemispheric non-corresponding vs. intrahemispheric 30
5.1 Face Network 32
5.2 Scene Network 34
5.3 Early Visual Network 36
6. Within-subject vs between-subject analysis 38

METHODS

1. Regions of Interest

1.1 StudyForrest dataset

Well established category-selective ROIs were identified for face (OFA, FFA, STS, & AMG), scene (OPA, PPA, & RSC), and dorsal (d) and ventral (v) early visual (V1d, V2d, V3d, V1v, V2v, & V3v) networks across the StudyForrest, Game of Thrones, and Human Connectome Project datasets.

For the StudyForrest dataset, face- and scene- selective regions were identified using a flood-fill algorithm to estimate 250 contiguous voxel clusters of high activation around a peak activating seed voxel in response to a relevant stimulus contrast (e.g., faces>scenes for face regions) in a localiser task specific to the dataset. Early visual regions were identified based on visual field masks generated by Wang *et al.* (2015). To align in size with other ROIs, early visual regions were restricted to the 250 contiguous voxel clusters around the peak probabilities of being a given visual area, based on full probabilities.

Regional seed MNI coordinates, actual cluster size, and Z-thresholds (face & scene regions) or full probability (early visual regions) are listed for each region within each hemisphere below (Table 1.1).

Table 1.1

MNI mm coordinates of seed voxels and thresholds or full probabilities of ROI clusters used for analysis of the StudyForrest dataset.

Region	Hemi	*x*	*y*	*z*	Cluster Size (vox)	Threshold *Z*/Full
Face Network						
OFA	L	-40.19	-79.94	-16.02	250	3.51
	R	38.26	-82.11	-7.86	250	4.22
FFA	L	-40.70	-53.74	-20.47	250	2.62
	R	38.13	-58.40	-15.95	250	3.52
STS	L	-46.15	-55.79	12.24	250	2.65
	R	53.66	-56.33	14.01	250	3.28

AMG	L	-17.79	-0.40	-17.58	251	2.14
	R	20.26	-2.40	-18.26	250	3.24
Scene Network						
OPA	L	-38.80	-84.00	17.66	250	3.74
	R	41.64	72.40	15.92	248	3.85
PPA	L	-22.55	-50.36	-8.31	250	4.13
	R	28.04	-46.71	-8.05	250	4.67
RSC	L	-16.43	-52.02	7.83	250	3.88
	R	8.64	-51.95	7.47	250	4.34
Early Visual						
V1d	L	-3.97	-101.76	-0.08	246	41.38
	R	10.61	-101.12	1.82	254	42.73
V2d	L	-8.48	-96.32	11.9	253	31.48
	R	9.91	-95.61	16.06	249	36.85
V3d	L	-21.68	-97.03	16.76	250	18.55
	R	26.93	-96.32	14.67	233	7.34
V1v	L	-7.74	-84.45	0.96	255	46.55
	R	9.57	-82.34	1.65	248	42.33
V2v	L	-6.05	-79.56	-10.32	253	37.41
	R	7.14	-79.56	-9.66	251	40.48
V3v	L	-14.74	-76.78	-12.41	244	14.7
	R	16.36	-77.28	13.45	251	15.7

Note. Full probabilities for early visual regions are expressed as minimum probability percentages.

1.2 Game of Thrones dataset

The same ROIs were established for the Game of Thrones dataset using the method described above, using a localiser specific to the dataset. Regional seed MNI coordinates, actual cluster size, and Z-thresholds (face & scene regions) are listed for each region within each hemisphere below (Table 1.2).

Regional seed MNI coordinates, cluster size, and full probabilities for early visual areas used in the Game of Thrones dataset were identical to those used in the StudyForrest dataset, so are not re-listed.

Table 1.2

MNI mm coordinates of seed voxels and thresholds of ROI clusters used for analysis of the Game of Thrones dataset.

Region	Hemi	*x*	*y*	*z*	Cluster Size (vox)	Threshold (*Z*)
Face Network						
OFA	L	-44	-79	-8	250	5.82
	R	47	-77	-4	250	6.47
FFA	L	-39	-51	-20	249	5.99
	R	42	-50	-18	250	6.26
STS	L	-44	-68	16	250	4.38
	R	50	-61	14	249	4.88
AMG	L	-20	-3	-15	250	3.17
	R	21	-4	-14	227	3.11
Scene Network						
OPA	L	-29	-90	16	250	7.54
	R	34	-83	17	250	7.95
PPA	L	-24	-52	-10	250	8.19
	R	26	-44	-9	250	8.91
RSC	L	-18	-55	8	250	5.05
	R	18	-52	10	250	5.98

1.3 Human Connectome Project dataset

With the Exception of the AMG, the Human connectome project's regions were registered as cortical-based surface vertices clusters. The average size of our 250 voxel volume clusters projected to the cortical surface was estimated at approximately 380 mm^2. As before, face- and scene- selective regions were identified using a flood-fill algorithm to estimate 380mm^2 contiguous vertices clusters of high activation around a peak activating seed vertex in response to a relevant stimulus contrast in a working-memory task used as a localiser. The amygdala was defined as a cluster of subcortical voxels in the same manner as described above for the StudyForrest and Game of Thrones datasets. Early visual regions were defined using the Benson Neuropythy pipeline, based on visual field masks generated by Wang *et al.* (2015).

Regional seed vertices or MNI coordinates, actual cluster size, and Z-thresholds are listed for each region within each hemisphere are listed below (Table 1.3). Early visual areas were generated from pre-existing Wang *et al.* (2015) masks, so are not listed here.

Table 1.3

MNI mm coordinates of seed voxels and thresholds of ROI clusters used analysis of the Human Connectome Project dataset.

Region	Hemi	*Seed vertex/voxel*			Cluster Size (mm^2/vox)	Threshold (*Z*)
Face Network						
OFA	L	-43.59	-72.86	8.54	380.56	5.47
	R	40.26	-81.71	-11.20	422.90	7.16
FFA	L	-39.85	-50.05	-19.35	380.08	7.18
	R	42.75	-49.05	-19.39	381.07	8.31
STS	L	-54.39	-44.68	9.89	376.59	3.32
	R	49.96	-39.79	9.03	379.22	5.24
AMG	L	-18.47	-5.61	-14.76	**253**	2.80
	R	17.45	-4.75	-14.76	**250**	2.69
Scene Network						
OPA	L	-30.36	-88.12	15.39	381.76	15.95
	R	35.18	-79.59	19.94	380.14	15.99
PPA	L	-30.27	-48.97	-8.13	380.26	15.64
	R	32.13	-46.45	-7.64	379.78	15.88

RSC	L	-17.68	-59.78	12.90	380.03	11.05
	R	18.56	-55.07	10.77	381.52	12.82

Note. Emboldened cluster sizes indicate sub-cortical ROI clusters generated as voxels rather than surface vertices clusters.

RESULTS

2. Within-subject interhemispheric corresponding vs. intrahemispheric

In this and subsequent supplementary sections, connectivity between corresponding regions (e.g., rOFA:lOFA) is abbreviated to 'interhemispheric connectivity'. Instances in which interhemispheric connectivity has been calculated between non-corresponding regions (e.g., rOFA:lFFA) is referred to with the 'non-corresponding' prefix.

For each region in each network, we compared the magnitude of interhemispheric correlation between corresponding regions (e.g., lOFA:rOFA) with the averaged correlation across all intrahemispheric pairings (e.g., lOFA:lFFA, lSTS, & lAMG), and with the highest-correlating intrahemispheric pairing with the same region (e.g., lOFA:lFFA) within each subject. These comparisons were repeated across all three datasets. Descriptive statistics are reported in Table 2.0, while inferential statistics are displayed in Sections 2.1 – 2.3 below.

Table 2.0

Within-subjects paired samples comparisons of corresponding interhemispheric, intrahemispheric average, and intrahemispheric highest means and standard deviations of Fishers' z correlations for three datasets.

Network & Region	Dataset & Connectivity Correlations								
	StudyForrest			Game of Thrones			H.C. Project		
	Inter	Intra A.	Intra H.	Inter	Intra A.	Intra H.	Inter	Intra A.	Intra H.
Face Selective Regions									
OFA	1.04(0.22)	0.53(0.13)	0.88(0.20)	1.15(0.23)	0.77(0.12)	1.06(0.18)	1.21(0.15)	0.58(0.09)	1.04(0.19)
FFA	1.05(0.16)	0.59(0.20)	0.88(0.20)	1.18(0.25)	0.78(0.12)	1.06(0.18)	1.07(0.22)	0.63(0.10)	1.04(0.19)
STS	0.89(0.27)	0.48(0.14)	0.65(0.15)	1.16(0.25)	0.70(0.13)	1.01(0.21)	0.99(0.19)	0.49(0.10)	0.69(0.17)
AMG	0.62(0.27)	0.34(0.14)	0.44(0.19)	0.83(0.26)	0.42(0.15)	0.51(0.18)	0.42(0.13)	0.30(0.10)	0.37(0.12)
Scene Selective Regions									
OPA	1.22(0.23)	0.57(0.13)	0.74(0.18)	1.23(0.23)	0.74(0.16)	0.90(0.17)	1.50(0.15)	0.76(0.11)	1.07(0.14)
PPA	1.08(0.11)	0.61(0.18)	0.74(0.18)	1.19(0.16)	0.88(0.15)	0.91(0.21)	1.25(0.14)	0.89(0.10)	1.07(0.14)
RSC	1.23(0.12)	0.53(0.14)	0.65(0.22)	1.30(0.20)	0.74(0.18)	0.91(0.21)	1.25(0.16)	0.61(0.11)	0.76(0.13)

Early Visual Regions									
V1d	1.01(0.32)	0.84(0.16)	1.08(0.17)	1.27(0.30)	1.21(0.15)	1.44(0.21)	1.62(0.20)	1.25(0.12)	1.55(0.22)
V2d	1.13(0.23)	0.77(0.18)	1.28(0.22)	1.37(0.23)	1.15(0.14)	1.50(0.23)	1.55(0.17)	1.19(0.15)	1.59(0.23)
V3d	1.16(0.21)	0.77(0.20)	1.28(0.22)	1.21(0.22)	1.04(0.14)	1.50(0.23)	1.48(0.18)	1.25(0.13)	1.59(0.23)
V1v	1.17(0.33)	0.91(0.16)	1.28(0.20)	1.41(0.26)	1.24(0.13)	1.53(0.21)	1.42(0.19)	1.26(0.12)	1.55(0.19)
V2v	1.23(0.20)	0.87(0.15)	1.41(0.26)	1.32(0.25)	1.19(0.15)	1.60(0.24)	1.41(0.18)	1.23(0.13)	1.59(0.18)
V3v	1.23(0.18)	0.90(0.16)	1.41(0.26)	1.33(0.20)	1.18(0.14)	1.60(0.24)	1.27(0.16)	1.17(0.13)	1.59(0.18)

2.1 Face Network

Interhemispheric correlations were of a significantly greater magnitude than all averaged intrahemispheric correlations (all $p \leq .001$). They were also significantly greater than all of the highest correlating intrahemispheric pairings (all $p \leq .046$)

Table 2.1

Within-subjects paired samples comparisons of corresponding interhemispheric, intrahemispheric average and intrahemispheric highest normalized time course of activity correlations (Zr) across face network ROIs for three datasets.

Network & Region	M_{diff}	95% CI	*t*	*p*	d_{avg}
**Interhemispheric vs. Intrahemispheric Avg.**					
StudyForrest					
OFA	0.51	[0.41, 0.61]	10.52	**<.001**	2.88
FFA	0.47	[0.38, 0.56]	11.24	**<.001**	2.56
STS	0.41	[0.28, 0.55]	6.69	**<.001**	1.92
AMG	0.28	[0.13, 0.43]	4.11	**.005**	1.30
Game of Thrones					
OFA	0.38	[0.32, 0.44]	12.12	**<.001**	2.07
FFA	0.40	[0.34, 0.46]	13.5	**<.001**	2.02
STS	0.47	[0.40, 0.53]	14.64	**<.001**	2.36
AMG	0.41	[0.35, 0.48]	12.57	**<.001**	1.97
Human Connectome Project					
OFA	0.63	[0.61, 0.66]	54.68	**<.001**	5.02
FFA	0.43	[0.41, 0.46]	32.75	**<.001**	2.55
STS	0.50	[0.48, 0.53]	35.14	**<.001**	3.32
AMG	0.12	[0.10, 0.13]	16.19	**<.001**	1.02
**Interhemispheric vs. Intrahemispheric Highest**					
StudyForrest					
OFA	0.17	[0.05, 0.29]	2.97	**.020**	0.80
FFA	0.18	[0.09, 0.27]	4.42	**.005**	0.99
STS	0.24	[0.11, 0.37]	3.90	**.006**	1.10
AMG	0.18	[0.00, 0.36]	2.19	**.046**	0.77
Game of Thrones					
OFA	0.09	[0.00, 0.17]	2.09	**.042**	0.42
FFA	0.13	[0.04, 0.21]	3.1	**.006**	0.57
STS	0.16	[0.08, 0.23]	4.07	**<.001**	0.68
AMG	0.32	[0.24, 0.40]	8.49	**<.001**	1.44
Human Connectome Project					
OFA	0.17	[0.14, 0.21]	10.98	**<.001**	1.02

FFA	0.03	[0.01, 0.06]	2.33	**.021**	0.17
STS	0.31	[0.27, 0.35]	15.28	**<.001**	1.68
AMG	0.05	[0.03, 0.06]	5.81	**<.001**	0.38

Note. For this, and for subsequent tables, *p*-values have been corrected for multiple comparisons using the Holm-Bonferroni method, and are emboldened if significant.

2.2 Scene Network

Interhemispheric correlations were of a significantly greater magnitude than all averaged intrahemispheric correlations (all $p < .001$).

They were also significantly greater than all of the highest correlating intrahemispheric pairings (all p < .001),

Table 2.2

Within-subjects paired samples comparisons of corresponding interhemispheric, intrahemispheric average, and intrahemispheric highest normalized time course of activity correlations (Zr) across scene network ROIs for three datasets.

Network & Region	M_{diff}	95% CI	*t*	*p*	d_{avg}
**Interhemispheric vs. Intrahemispheric Avg.**					
StudyForrest					
OPA	0.65	[0.54, 0.76]	12.53	**<.001**	3.51
PPA	0.47	[0.35, 0.59]	8.66	**<.001**	3.19
RSC	0.7	[0.61, 0.8,]	15.31	**<.001**	5.22
Game of Thrones					
OPA	0.48	[0.42, 0.55]	15.72	**<.001**	2.46
PPA	0.31	[0.27, 0.35]	14.80	**<.001**	2.02
RSC	0.56	[0.51, 0.60]	25.74	**<.001**	2.94
Human Connectome Project					
OPA	0.74	[0.72 0.76]	69.44	**<.001**	5.78
PPA	0.36	[0.34 0.38]	40.42	**<.001**	3.06
RSC	0.64	[0.62 0.66]	72.26	**<.001**	4.71
**Interhemispheric vs. Intrahemispheric Highest**					
StudyForrest					
OPA	0.49	[0.35, 0.62]	7.64	**<.001**	2.38
PPA	0.34	[0.23, 0.44]	6.88	**<.001**	2.26
RSC	0.58	[0.45, 0.71]	9.49	**<.001**	3.31
Game of Thrones					
OPA	0.33	[0.26, 0.40]	9.49	**<.001**	1.64
PPA	0.28	[0.22, 0.33]	9.99	**<.001**	1.49
RSC	0.38	[0.33, 0.43]	15.23	**<.001**	1.91
Human Connectome Project					
OPA	0.43	[0.41 0.45]	36.97	**<.001**	2.96
PPA	0.19	[0.16 0.21]	14.52	**<.001**	1.31
RSC	0.49	[0.47 0.51]	52.1	**<.001**	3.4

2.3 Early Visual Network

Interhemispheric correlations were of a significantly greater magnitude than all but two of the averaged intrahemispheric correlations (all $p \leq .038$), only reaching a significant trend for higher interhemispheric connectivity in the V1d in the StudyForrest [$t(14) = 2.65$, $p = .114$, $d_{avg} = 0.68$]; and Game of Thrones [$t(44) = 1.96$, $p = .057$, $d_{avg} = 0.29$] datasets.

However, Interhemispheric correlations were significantly lower than all of the highest correlating intrahemispheric pairings in the Game of Thrones and Human Connectome Project (all $p \leq .049$). While early visual region correlations were also directed towards lower interhemispheric correlations in the StudyForrest dataset, the difference did not reach significance across any region (p ≥ .056).

Table 2.3

Within-subjects paired samples comparisons of corresponding interhemispheric, intrahemispheric average, and intrahemispheric highest normalized time course of activity correlations (Zr) across early visual network ROIs for three datasets.

Network & Region	M_{diff}	95% CI	t	p	d_{avg}
**Interhemispheric vs. Intrahemispheric Avg.**					
StudyForrest					
V1d	0.17	[0.03, 0.31]	2.65	.114	0.68
V2d	0.36	[0.23, 0.50]	5.64	**<.001**	1.76
V3d	0.39	[0.24, 0.54]	5.70	**<.001**	1.90
V1v	0.26	[0.13, 0.40]	4.19	**.008**	1.02
V2v	0.36	[0.27, 0.45]	8.61	**<.001**	2.09
V3v	0.33	[0.22, 0.43]	6.75	**<.001**	1.93
Game of Thrones					
V1d	0.07	[-0.00, 0.14]	1.96	.057	0.29
V2d	0.22	[0.16, 0.27]	7.96	**<.001**	1.15
V3d	0.17	[0.12, 0.22]	6.52	**<.001**	0.90
V1v	0.18	[0.12, 0.23]	6.19	**<.001**	0.85
V2v	0.14	[0.08, 0.19]	4.99	**<.001**	0.67
V3v	0.15	[0.10, 0.19]	6.37	**<.001**	0.84
Human Connectome Project					
V1d	0.37	[0.34, 0.39]	30.08	**<.001**	2.25
V2d	0.36	[0.34, 0.39]	27.95	**<.001**	2.22
V3d	0.23	[0.21, 0.25]	25.7	**<.001**	1.48
V1v	0.15	[0.13, 0.18]	11.63	**<.001**	0.96
V2v	0.18	[0.15, 0.20]	14.93	**<.001**	1.14

V3v	0.10	[0.08, 0.12]	10.26	**<.001**	0.67
**Interhemispheric vs. Intrahemispheric Highest**					
StudyForrest					
V1d	-0.07	[-0.27, 0.13]	-0.76	.460	0.28
V2d	-0.15	[-0.28, -0.02]	-2.49	.130	0.65
V3d	-0.12	[-0.23, -0.00]	-2.15	.152	0.54
V1v	-0.10	[-0.23, 0.02]	-1.80	.188	0.39
V2v	-0.18	[-0.30, -0.06]	-3.11	.056	0.78
V3v	-0.18	[-0.35, -0.01]	-2.29	.152	0.79
Game of Thrones					
V1d	-0.17	[-0.27, -0.06]	-3.11	**.006**	0.65
V2d	-0.13	[-0.20, -0.07]	-4.07	**<.001**	0.57
V3d	-0.29	[-0.36, -0.22]	-8.32	**<.001**	1.29
V1v	-0.12	[-0.18, -0.05]	-3.49	**.003**	0.49
V2v	-0.28	[-0.35, -0.20]	-7.42	**<.001**	1.11
V3v	-0.27	[-0.34, -0.20]	-7.87	**<.001**	1.20
Human Connectome Project					
V1d	0.07	[0.04 0.11]	4.08	**<.001**	0.35
V2d	-0.03	[-0.07 -0.00]	-1.98	**.049**	0.17
V3d	-0.11	[-0.13 -0.08]	-8.00	**<.001**	0.52
V1v	-0.13	[-0.17 -0.09]	-6.84	**<.001**	0.67
V2v	-0.18	[-0.21 -0.15]	-11.70	**<.001**	1.02
V3v	-0.32	[-0.35 -0.30]	-27.42	**<.001**	1.90

3. Within-subject interhemispheric non-corresponding vs. intrahemispheric

For each region in each network, we also compared the magnitude of non-corresponding interhemispheric correlations with intrahemispheric correlations within each subject.

First, comparisons were made between correlations averaged across all non-corresponding interhemispheric pairings (e.g., lOFA:rFFA, rSTS, & rAMG) and the averaged correlation across all intrahemispheric pairings

Second, comparisons were made between the highest-correlating non-corresponding interhemispheric pairing (e.g., lOFA:rFFA) and the highest-correlating intrahemispheric pairing. These comparisons were repeated across all three datasets. Descriptive statistics are reported in Table 3.0, while inferential statistics are displayed in Sections 3.1 – 3.3 below.

Table 3.0

Within-subjects paired samples comparisons of non-corresponding interhemispheric average, interhemispheric highest, intrahemispheric average, and intrahemispheric highest means and (standard deviations) of Fishers' z correlations for three datasets.

Network & Region	Dataset & Connectivity Correlations											
	StudyForrest				Game of Thrones				H.C. Project			
	Inter Avg.	Inter High.	Intra Avg.	Intra High.	Inter Avg.	Inter High.	Intra Avg.	Intra High.	Inter Avg.	Inter High.	Intra Avg.	Intra High.
Face Selective Regions												
OFA	0.55 (0.14)	0.53 (0.13)	0.76 (0.20)	0.88 (0.20)	0.74 (0.14)	0.77 (0.12)	1.00 (0.21)	1.06 (0.18)	0.54 (0.11)	0.58 (0.09)	0.94 (0.16)	1.04 (0.19)
FFA	0.56 (0.21)	0.59 (0.20)	0.76 (0.20)	0.88 (0.20)	0.73 (0.16)	0.78 (0.12)	1.00 (0.21)	1.06 (0.18)	0.65 (0.12)	0.63 (0.10)	0.94 (0.16)	1.04 (0.19)
STS	0.43 (0.16)	0.48 (0.14)	0.57 (0.21)	0.65 (0.15)	0.64 (0.17)	0.70 (0.13)	0.83 (0.23)	1.01 (0.21)	0.44 (0.12)	0.49 (0.10)	0.67 (0.18)	0.69 (0.17)
AMG	0.31 (0.15)	0.34 (0.14)	0.42 (0.24)	0.44 (0.19)	0.40 (0.15)	0.42 (0.15)	0.50 (0.21)	0.51 (0.18)	0.28 (0.11)	0.30 (0.10)	0.36 (0.11)	0.37 (0.12)
Scene Selective Regions												
OPA	0.60 (0.11)	0.57 (0.13)	0.67 (0.19)	0.74 (0.18)	0.72 (0.17)	0.74 (0.16)	0.83 (0.17)	0.90 (0.17)	0.77 (0.11)	0.76 (0.11)	1.02 (0.14)	1.07 (0.14)
PPA	0.54 (0.15)	0.61 (0.18)	0.67 (0.19)	0.74 (0.18)	0.86 (0.16)	0.88 (0.15)	0.89 (0.20)	0.91 (0.21)	0.88 (0.10)	0.89 (0.10)	1.02 (0.14)	1.07 (0.14)

RSC	0.45<br>(0.18)	0.53<br>(0.14)	0.53<br>(0.10)	0.65<br>(0.22)	0.67<br>(0.21)	0.74<br>(0.18)	0.89<br>(0.20)	0.91<br>(0.21)	0.55<br>(0.12)	0.61<br>(0.11)	0.80<br>(0.13)	0.76<br>(0.13)
Early Visual Regions												
V1d	0.76<br>(0.20)	0.84<br>(0.16)	0.85<br>(0.23)	1.08<br>(0.17)	1.08<br>(0.16)	1.21<br>(0.15)	1.22<br>(0.22)	1.44<br>(0.21)	1.12<br>(0.13)	1.25<br>(0.12)	1.38<br>(0.18)	1.55<br>(0.22)
V2d	0.67<br>(0.16)	0.77<br>(0.18)	1.00<br>(0.20)	1.28<br>(0.22)	1.00<br>(0.18)	1.15<br>(0.14)	1.24<br>(0.18)	1.50<br>(0.23)	1.08<br>(0.15)	1.19<br>(0.15)	1.38<br>(0.18)	1.59<br>(0.23)
V3d	0.71<br>(0.18)	0.77<br>(0.20)	1.00<br>(0.20)	1.28<br>(0.22)	0.98<br>(0.15)	1.04<br>(0.14)	1.24<br>(0.18)	1.50<br>(0.23)	1.16<br>(0.13)	1.25<br>(0.13)	1.35<br>(0.17)	1.59<br>(0.23)
V1v	0.79<br>(0.21)	0.91<br>(0.16)	1.10<br>(0.31)	1.28<br>(0.20)	1.06<br>(0.15)	1.24<br>(0.13)	1.23<br>(0.27)	1.53<br>(0.21)	1.10<br>(0.13)	1.26<br>(0.12)	1.26<br>(0.15)	1.55<br>(0.19)
V2v	0.70<br>(0.18)	0.87<br>(0.15)	1.10<br>(0.31)	1.41<br>(0.26)	1.03<br>(0.18)	1.19<br>(0.15)	1.24<br>(0.26)	1.60<br>(0.24)	1.09<br>(0.13)	1.23<br>(0.13)	1.26<br>(0.15)	1.59<br>(0.18)
V3v	0.76<br>(0.16)	0.90<br>(0.16)	1.09<br>(0.21)	1.41<br>(0.26)	1.02<br>(0.14)	1.18<br>(0.14)	1.24<br>(0.26)	1.60<br>(0.24)	1.03<br>(0.13)	1.17<br>(0.13)	1.23<br>(0.15)	1.59<br>(0.18)

3.1 Face Network

Comparison of averaged non-corresponding interhemispheric and intrahemispheric correlations showed an overall trend towards greater correlations between intrahemispheric regions. This was significant across the majority of face regions in the Game of Thrones and Human Connectome Project datasets (all $p \leq .020$) with the exception of the Game of Thrones OFA [$t(44) = -2.18$, $p = .136$, $d_{avg} = 0.22$], and the Human Connectome Project FFA [$t(173) = 4.04$, $p < .001$, $d_{avg} = 0.18$]. No significant differences were found in the StudyForrest dataset ($p \leq .132$).

Comparisons of highest correlating pairings showed a similar pattern, with an overall direction towards stronger intrahemispheric connectivity. However this only reached significance across all regions in the Human Connectome Project dataset (all $p \leq .030$), but failed to reach significance for all regions in the StudyForrest Dataset (all $p \geq .072$), and all but the FFA [$t(44) = -5.87$, $p < .001$, $d_{avg} = 0.80$] in the Game of Thrones Dataset.

Table 3.1

Within-subjects paired samples comparisons of non-corresponding interhemispheric average, interhemispheric highest, intrahemispheric average, and intrahemispheric highest normalized time course of activity correlations (Zr) across face network ROIs for three datasets.

Network & Region	M_{diff}	95% CI	*t*	*p*	d_{avg}
**NC Interhemispheric Avg. vs. Intrahemispheric Avg.**					
StudyForrest					
OFA	0.02	[-0.02, 0.06]	0.97	.668	0.14
FFA	-0.03	[-0.07, 0.00]	-1.92	.375	0.16
STS	-0.05	[-0.09, -0.01]	-2.58	.132	0.30
AMG	-0.04	[-0.08, 0.00]	-1.92	.375	0.26
Game of Thrones					
OFA	-0.03	[-0.06, -0.00]	-2.18	.136	0.22
FFA	-0.05	[-0.08, -0.02]	-3.54	**.007**	0.34
STS	-0.05	[-0.08, -0.02]	-3.60	**.007**	0.36
AMG	-0.02	[-0.04, -0.01]	-3.03	**.020**	0.15
Human Connectome Project					

OFA	-0.03	[-0.02, -0.06]	-7.62	**<.001**	0.32
FFA	0.02	[0.03, 0.02]	4.04	**<.001**	0.18
STS	-0.05	[-0.03, -0.07]	-9.72	**<.001**	0.46
AMG	-0.02	[-0.01, -0.04]	-7.08	**<.001**	0.19
**NC Interhemispheric Highest vs. Intrahemispheric Highest**					
StudyForrest					
OFA	-0.12	[-0.21, -0.04]	-3.03	.072	0.60
FFA	-0.12	[-0.21, -0.04]	-3.03	.072	0.60
STS	-0.08	[-0.21, 0.05]	-1.36	.585	0.43
AMG	-0.02	[-0.07, 0.03]	-1.00	.668	0.10
Game of Thrones					
OFA	-0.06	[-0.12, 0.00]	-2.01	.150	0.30
FFA	-0.06	[-0.12, 0.00]	-2.01	.150	0.30
STS	-0.18	[-0.24, -0.12]	-5.87	**<.001**	0.80
AMG	-0.01	[-0.04, 0.02]	-0.64	.524	0.05
Human Connectome Project					
OFA	-0.09	[-0.12, -0.07]	-6.81	**<.001**	0.29
FFA	-0.09	[-0.12, -0.07]	-6.81	**<.001**	0.29
STS	-0.02	[-0.04, -0.00]	-2.18	**.030**	0.36
AMG	-0.01	[-0.02, -0.01]	-3.79	**<.001**	0.13

3.2 Scene Network

Comparison of averaged non-corresponding interhemispheric and intrahemispheric correlations showed no significant differences between interhemispheric and intrahemispheric average correlations across all groups for the OPA ($p \geq .144$). However, significantly greater intrahemispheric correlations across the RSC and PPA across all datasets (all $p \leq .005$), except the Game of Thrones PPA, which did not reach significance [$t(44) = -2.16$, $p = .144$, $d_{avg} = 0.12$].

This direction was maintained across the majority of comparisons between highest correlating pairings, however failed to reach significance in both the StudyForrest and Game of Thrones datasets (all $p \geq .308$) with the exception of the Game of Thrones OPA [$t(44) = -3.88$, $p < .001$, $d_{avg} = 0.39$]. For the Human Connectome project dataset, intrahemispheric correlations were significantly greater for both the OPA and PPA (both $p < .001$). Contrastingly, the interhemispheric correlation between rRSC:lPPA was greater than any intrahemispheric connection with the RSC [$t(173) = 6.52$, $p < .001$, $d_{avg} = 0.24$].

Table 3.2

Within-subjects paired samples comparisons of non-corresponding interhemispheric average, interhemispheric highest, intrahemispheric average, and intrahemispheric highest normalized time course of activity correlations (Zr) across scene network ROIs for three datasets.

Network & Region	M_{diff}	95% CI	*t*	*p*	d_{avg}
**NC Interhemispheric Avg. vs. Intrahemispheric Avg.**					
StudyForrest					
OPA	0.03	[-0.00, 0.05]	2.03	.216	0.21
PPA	-0.07	[-0.10, -0.03]	-3.98	**.006**	0.41
RSC	-0.08	[-0.11, -0.04]	-4.25	**.006**	0.46
Game of Thrones					
OPA	-0.02	[-0.04, -0.00]	-2.13	.144	0.14
PPA	-0.02	[-0.04, -0.00]	-2.16	.144	0.12
RSC	-0.07	[-0.09, -0.05]	-6.93	**<.001**	0.35
Human Connectome Project					
OPA	0.00	[-0.01, 0.01]	0.74	.462	0.03

PPA	-0.02	[-0.04, -0.03]	-5.15	**<.001**	0.16
RSC	-0.07	[-0.09, -0.06]	-22.02	**<.001**	0.58
**NC Interhemispheric Highest vs. Intrahemispheric Highest**					
StudyForrest					
OPA	-0.07	[-0.14, 0.00]	-2.05	.216	0.38
PPA	-0.07	[-0.14, 0.00]	-2.05	.216	0.38
RSC	-0.11	[-0.23, 0.00]	-2.10	.216	0.68
Game of Thrones					
OPA	-0.07	[-0.10, -0.03]	-3.88	**<.001**	0.39
PPA	-0.02	[-0.06, 0.01]	-1.45	.308	0.11
RSC	-0.02	[-0.06, 0.01]	-1.45	.308	0.11
Human Connectome Project					
OPA	-0.05	[-0.07, -0.04]	-7.52	**<.001**	0.37
PPA	-0.05	[-0.07, -0.04]	-7.52	**<.001**	0.37
RSC	0.03	[0.02, 0.04]	6.52	**<.001**	0.24

3.3 *Early Visual Network*

Comparison of averaged non-corresponding interhemispheric and intrahemispheric correlations showed significantly greater intrahemispheric correlations across all regions and datasets (all $p \leq .048$), except the StudyForrest V1d, which only reached a significant trend in this direction [$t(173) = -2.13$, $p = .051$, $d_{avg} = 0.41$].

This finding was repeated in comparisons of highest correlating pairing (all $p \leq .042$).

Table 3.3

Within-subjects paired samples comparisons of non-corresponding interhemispheric average, interhemispheric highest, intrahemispheric average, and intrahemispheric highest normalized time course of activity correlations (Zr) across early visual network ROIs for three datasets.

Network & Region	M_{diff}	95% CI	*t*	*p*	d_{avg}
**NC Interhemispheric Avg. vs. Intrahemispheric Avg.**					
StudyForrest					
V1d	-0.07	[-0.15, 0.00]	-2.13	.051	0.41
V2d	-0.10	[-0.15, -0.05]	-4.07	**.007**	0.58
V3d	-0.06	[-0.11, -0.01]	-2.55	**.046**	0.32
V1v	-0.12	[-0.21, -0.04]	-3.24	**.030**	0.66
V2v	-0.17	[-0.22, -0.12]	-7.21	**<.001**	1.05
V3v	-0.14	[-0.20, -0.08]	-5.09	**<.001**	0.87
Game of Thrones					
V1d	-0.12	[-0.14, -0.10]	-7.84	**<.001**	0.80
V2d	-0.16	[-0.17, -0.15]	-12.14	**<.001**	0.96
V3d	-0.06	[-0.08, -0.04]	-4.68	**<.001**	0.41
V1v	-0.17	[-0.18, -0.15]	-11.98	**<.001**	1.23
V2v	-0.16	[-0.18, -0.15]	-10.81	**<.001**	0.96
V3v	-0.16	[-0.19, -0.13]	-16.93	**<.001**	1.10
Human Connectome Project					
V1d	-0.13	[-0.14 -0.12]	-33.23	**<.001**	1.06
V2d	-0.11	[-0.11 -0.10]	-22.14	**<.001**	0.75
V3d	-0.10	[-0.11 -0.09]	-22.86	**<.001**	0.77
V1v	-0.16	[-0.16 -0.15]	-36.78	**<.001**	1.33
V2v	-0.14	[-0.15 -0.14]	-31.72	**<.001**	1.09
V3v	-0.14	[-0.15 -0.14]	-29.81	**<.001**	1.03
**NC Interhemispheric Highest vs. Intrahemispheric Highest**					
StudyForrest					
V1d	-0.23	[-0.39, -0.07]	-3.12	**.030**	1.16
V2d	-0.28	[-0.37, -0.18]	-6.24	**<.001**	1.31
V3d	-0.28	[-0.37, -0.18]	-6.24	**<.001**	1.31
V1v	-0.18	[-0.31, -0.04]	-2.79	**.042**	0.68
V2v	-0.31	[-0.48, -0.14]	-3.91	**.012**	1.08

V3v	-0.32	[-0.41, -0.24]	-8.22	**<.001**	1.37
Game of Thrones					
V1d	-0.22	[-0.28, -0.15]	-6.78	**<.001**	1.01
V2d	-0.26	[-0.32, -0.21]	-9.81	**<.001**	1.27
V3d	-0.26	[-0.32, -0.21]	-9.81	**<.001**	1.27
V1v	-0.30	[-0.36, -0.23]	-9.27	**<.001**	1.23
V2v	-0.36	[-0.42, -0.29]	-10.87	**<.001**	1.43
V3v	-0.36	[-0.42, -0.29]	-10.87	**<.001**	1.43
Human Connectome Project					
V1d	-0.17	[-0.19, -0.15]	-16.28	**<.001**	0.85
V2d	-0.21	[-0.24, -0.17]	-12.88	**<.001**	1.01
V3d	-0.24	[-0.26, -0.22]	-20.24	**<.001**	1.20
V1v	-0.28	[-0.31, -0.26]	-24.63	**<.001**	1.65
V2v	-0.33	[-0.35, -0.30]	-26.36	**<.001**	1.99
V3v	-0.36	[-0.38, -0.34]	-36.55	**<.001**	2.21

4. Between-subject interhemispheric corresponding vs. intrahemispheric

For between-subject comparisons of each region in each network, we generated between-subjects measures of interhemispheric activity by correlating time-courses of activity for individual corresponding regions from two subjects' different hemispheres. (e.g., lOFA from P1:rOFA from P2). This was repeated and averaged all possible subject pairings for each individual subject (e.g., P1:P2-P14). Between-subjects measures of intrahemispheric connectivity were generated in the same manner, only for regions within two subjects' matching hemispheres (e.g., lOFA from P1:lFFA from P2), again being repeated and averaged all possible subject pairings for each individual subject. As for within-subject comparisons, interhemispheric correlations for each region were compared with the averaged correlation across all intrahemispheric pairings within a network (e.g., lOFA:lFFA, lSTS, & lAMG), and with the highest-correlating intrahemispheric pairing among these (e.g., lOFA:lFFA). These comparisons were repeated across all three datasets. Descriptive statistics are reported in Table 4.0, while inferential statistics are displayed in Sections 4.1 – 4.3 below.

Table 4.0

Between-subjects paired samples comparisons of corresponding interhemispheric, intrahemispheric average, and intrahemispheric highest means and (standard deviations) of Fishers' z correlations for three datasets.

Network & Region	Dataset & Connectivity Correlations								
	StudyForrest			Game of Thrones			H.C. Project		
	Inter	Intra A.	Intra H.	Inter	Intra A.	Intra H.	Inter	Intra A.	Intra H.
Face Selective Regions									
OFA	0.26(0.04)	0.13(0.02)	0.22(0.03)	0.28(0.06)	0.18(0.04)	0.30(0.06)	0.52(0.09)	0.26(0.05)	0.45(0.08)
FFA	0.17(0.03)	0.13(0.02)	0.22(0.03)	0.21(0.05)	0.16(0.03)	0.25(0.05)	0.41(0.08)	0.27(0.05)	0.45(0.08)
STS	0.18(0.05)	0.11(0.02)	0.19(0.05)	0.32(0.08)	0.17(0.04)	0.30(0.06)	0.35(0.07)	0.20(0.04)	0.30(0.06)
AMG	0.03(0.01)	0.05(0.01)	0.06(0.01)	0.04(0.02)	0.05(0.02)	0.08(0.03)	0.07(0.02)	0.13(0.03)	0.16(0.04)

Scene Selective Regions									
OPA	0.38(0.06)	0.22(0.04)	0.33(0.04)	0.26(0.06)	0.18(0.05)	0.26(0.06)	0.43(0.08)	0.30(0.06)	*0.42(0.07)*
PPA	0.23(0.05)	0.22(0.04)	0.33(0.04)	0.30(0.06)	0.22(0.05)	0.26(0.06)	0.44(0.08)	0.34(0.06)	*0.42(0.07)*
RSC	0.14(0.04)	0.15(0.03)	0.19(0.04)	0.18(0.06)	0.17(0.05)	0.23(0.06)	0.23(0.06)	0.23(0.05)	*0.29(0.06)*
Early Visual Regions									
V1d	0.23(0.04)	0.23(0.03)	0.31(0.05)	0.33(0.08)	0.31(0.07)	0.36(0.08)	0.45(0.09)	0.40(0.09)	0.46(0.10)
V2d	0.30(0.04)	0.22(0.03)	0.31(0.05)	0.30(0.08)	0.29(0.06)	0.34(0.09)	0.44(0.10)	0.39(0.08)	0.46(0.10)
V3d	0.26(0.05)	0.24(0.04)	0.30(0.04)	0.26(0.07)	0.27(0.06)	0.32(0.08)	0.41(0.09)	0.40(0.08)	0.43(0.09)
V1v	0.27(0.04)	0.24(0.03)	0.32(0.05)	0.36(0.08)	0.32(0.06)	0.36(0.08)	0.41(0.09)	0.40(0.09)	0.45(0.09)
V2v	0.27(0.04)	0.23(0.03)	0.34(0.04)	0.27(0.06)	0.28(0.06)	0.33(0.07)	0.43(0.09)	0.40(0.08)	0.46(0.10)
V3v	0.33(0.04)	0.26(0.03)	0.34(0.04)	0.30(0.06)	0.29(0.06)	0.34(0.07)	0.42(0.09)	0.39(0.08)	0.46(0.10)

4.1 Face Network

For the OFA, FFA, and STS, interhemispheric correlations were of a significantly greater magnitude than all averaged intrahemispheric correlations (all $p < .001$). Contrastingly, for the AMG, averaged intrahemispheric correlations were of a significantly greater magnitude than all interhemispheric correlations (all $p < .001$).

In comparisons of interhemispheric pairings with the highest correlating intrahemispheric pairings, the OFA showed an inconsistent pattern, with higher interhemispheric correlations in the StudyForrest dataset [$t(14) = 5.46$, $p < .001$, $d_{avg} = 1.06$] and Human Connectome Project dataset [$t(173) = 21.31$, $p < .001$, $d_{avg} = 0.82$]; but higher intrahemispheric correlations in the Game of Thrones dataset [$t(44) = -3.03$, $p = .008$, $d_{avg} = 0.37$]. More consistently, the FFA showed a direction towards higher intrahemispheric correlations. This was significant across all datasets (all $p < .001$). Contrastingly, The STS showed higher interhemispheric correlations in the Human Connectome Project dataset [$t(173) = 0.05$, $p < .001$, $d_{\text{avg}} = 0.79$]; but no significant differences in the StudyForrest and Game of Thrones datasets ($p \geq .056$). The AMG showed a consistent pattern of higher intrahemispheric correlations across all datasets (all $p < .001$).

Table 4.1

Between-subjects paired samples comparisons of corresponding interhemispheric, intrahemispheric average, and intrahemispheric highest normalized time course of activity correlations (Zr) across face network ROIs for three datasets.

Network & Region	M_{diff}	95% CI	t	p	d_{avg}
**Interhemispheric vs. Intrahemispheric Avg.**					
StudyForrest					
OFA	0.13	[0.11, 0.15]	15.84	**<.001**	3.86
FFA	0.05	[0.04, 0.06]	12.23	**<.001**	1.72
STS	0.07	[0.05, 0.09]	7.90	**<.001**	1.95
AMG	-0.02	[-0.02, -0.01]	-10.32	**<.001**	2.23

Game of Thrones					
OFA	0.10	[0.09, 0.11]	18.86	**<.001**	1.96
FFA	0.05	[0.04, 0.06]	13.03	**<.001**	1.15
STS	0.16	[0.14, 0.17]	19.36	**<.001**	2.42
AMG	-0.01	[-0.02, -0.01]	-6.17	**<.001**	0.77
Human Connectome Project					
OFA	0.26	[0.25, 0.27]	66.94	**<.001**	3.7
FFA	0.14	[0.13, 0.14]	50.87	**<.001**	2.19
STS	0.15	[0.14, 0.15]	40.48	**<.001**	2.54
AMG	-0.05	[-0.05, -0.05]	-61.61	**<.001**	2.18
**Interhemispheric vs. Intrahemispheric Highest**					
StudyForrest					
OFA	0.04	[0.03, 0.06]	5.46	**<.001**	1.06
FFA	-0.05	[-0.06, -0.03]	-7.58	**<.001**	1.35
STS	-0.01	[-0.04, 0.02]	-0.58	.571	0.18
AMG	-0.03	[-0.04, -0.03]	-13.67	**<.001**	3.33
Game of Thrones					
OFA	-0.02	[-0.04, -0.01]	-3.03	**.008**	0.37
FFA	-0.04	[-0.04, -0.03]	-9.37	**<.001**	0.67
STS	0.02	[-0.00, 0.04]	1.96	.056	0.24
AMG	-0.04	[-0.04, -0.03]	-10.52	**<.001**	1.43
Human Connectome Project					
OFA	0.07	[0.06, 0.08]	21.31	**<.001**	0.82
FFA	-0.04	[-0.05, -0.04]	-12.58	**<.001**	0.54
STS	0.05	[0.04, 0.06]	12.09	**<.001**	0.79
AMG	-0.09	[-0.09, -0.09]	-52.39	**<.001**	3.05

4.2 Scene Network

For the OPA and PPA, interhemispheric correlations were of a significantly greater magnitude than all averaged intrahemispheric correlations (all $p \leq .010$). the RSC was less consistent, showing greater averaged intrahemispheric correlations in the StudyForrest dataset [$t(14) = -$ -4.01, $p = .002$, $d_{avg} = 0.40$], greater interhemispheric correlations in the Game of Thrones dataset [$t(44) = 3.97$, $p < .001$, $d_{avg} = 0.24$], and no significant differences between correlations in the Human Connectome Project dataset [$t(173) = -0.08$, $p = .937$, $d_{avg} = 0.00$].

In comparisons of interhemispheric pairings with the highest correlating intrahemispheric pairings, the OPA showed consistently stronger interhemispheric correlations in the StudyForrest and Human Connectome Project Datasets (p < .001), but not the Game of Thrones Dataset [$t(44) = 0.00$, $p = .313$, $d_{avg} = 0.07$. The PPA was also inconsistent, showing greater intrahemispheric correlations in the StudyForrest dataset [$t(14) = -10.27$, $p < .001$, d_{avg} $= 2.18$, but greater interhemispheric correlations in the Game of Thrones and Human Connectome Project Datasets (both $p < .001$). The RSC showed stronger intrahemispheric correlations across all datasets (all $p < .001$).

Table 4.2

Between-subjects paired samples comparisons of corresponding interhemispheric, intrahemispheric average, and intrahemispheric highest normalized time course of activity correlations (Zr) across scene network ROIs for three datasets.

Network & Region	M_{diff}	95% CI	*t*	*p*	d_{avg}
**Interhemispheric vs. Intrahemispheric Avg.**					
StudyForrest					
OPA	0.17	[0.15, 0.18]	18.82	**<.001**	3.36
PPA	0.01	[0.00, 0.02]	2.99	**.010**	0.29
RSC	-0.01	[-0.02, -0.01]	-4.01	**.002**	0.40
Game of Thrones					
OPA	0.07	[0.06, 0.08]	17.01	**<.001**	1.34
PPA	0.08	[0.07, 0.08]	27.88	**<.001**	1.33
RSC	0.01	[0.01, 0.02]	3.97	**<.001**	0.24

Human Connectome Project					
OPA	0.13	[0.12, 0.13]	55.62	**<.001**	1.87
PPA	0.11	[0.10, 0.11]	68.83	**<.001**	1.50
RSC	-0.00	[-0.00, 0.00]	-0.08	.937	0.00
**Interhemispheric vs. Intrahemispheric Highest**					
StudyForrest					
OPA	0.06	[0.04, 0.07]	7.99	**<.001**	1.09
PPA	-0.10	[-0.12, -0.08]	-10.27	**<.001**	2.18
RSC	-0.05	[-0.06, -0.04]	-15.36	**<.001**	1.14
Game of Thrones					
OPA	0.00	[-0.01, 0.00]	-1.02	.313	0.07
PPA	0.03	[0.02, 0.04]	6.97	**<.001**	0.57
RSC	-0.05	[-0.05, -0.04]	-14.6	**<.001**	0.76
Human Connectome Project					
OPA	0.01	[0.01, 0.02]	8.78	**<.001**	0.18
PPA	0.02	[0.02, 0.03]	11.5	**<.001**	0.29
RSC	-0.06	[-0.06, -0.05]	-33.37	**<.001**	0.93

4.3 Early Visual Network

The majority of regions in the early visual network showed significantly greater interhemispheric correlations than averaged intrahemispheric correlations (all $p \leq .005$). The exceptions to this were the V1d in the StudyForrest Dataset [$t(14) = 2.02$, $p = .189$, $d_{avg} = 0.23$], and the V3d and V2v in the Game of Thrones dataset, which showed higher averaged intrahemispheric correlations (both $p < .001$).

However, with only three exceptions, Interhemispheric correlations were lower in magnitude than those of the highest correlating intrahemispheric pairings (all $p < .001$). These exceptions were V2d and V3v in the StudyForrest dataset, and V1v in the Game of Thrones dataset. While all of these maintained the same direction, they did not reach significance (all $p \geq .252$).

Table 4.3

Between-subjects paired samples comparisons of corresponding interhemispheric, intrahemispheric average, and intrahemispheric highest normalized time course of activity correlations (Zr) across early visual network ROIs for three datasets.

Network & Region	M_{diff}	95% CI	t	p	d_{avg}
**Interhemispheric vs. Intrahemispheric Avg.**					
StudyForrest					
V1d	0.01	[-0.00, 0.02]	2.02	.189	0.23
V2d	0.08	[0.07, 0.10]	13.44	**<.001**	2.52
V3d	0.02	[0.01, 0.03]	4.61	**<.001**	0.57
V1v	0.03	[0.02, 0.04]	5.77	**<.001**	0.72
V2v	0.04	[0.03, 0.06]	7.11	**<.001**	1.20
V3v	0.07	[0.06, 0.09]	15.13	**<.001**	2.14
Game of Thrones					
V1d	0.02	[0.02, 0.03]	8.20	**<.001**	0.31
V2d	0.01	[0.00. 0.02]	3.51	**.003**	0.15
V3d	-0.02	[-0.02, -0.01]	-5.76	**<.001**	0.24
V1v	0.04	[0.04, 0.05]	14.13	**<.001**	0.59
V2v	-0.01	[-0.02, -0.01]	-3.88	**<.001**	0.20
V3v	0.01	[-0.00, 0.01]	2.63	**.024**	0.13
Human Connectome Project					
V1d	0.04	[0.04, 0.04]	35.28	**<.001**	0.47
V2d	0.05	[0.05, 0.06]	34.58	**<.001**	0.61
V3d	0.01	[0.01, 0.01]	9.66	**<.001**	0.11
V1v	0.00	[0.00, 0.00]	2.87	**.005**	0.03
V2v	0.03	[0.03, 0.03]	27.17	**<.001**	0.36

V3v	0.03	[0.03, 0.03]	25.70	**<.001**	0.37
**Interhemispheric vs. Intrahemispheric Highest**					
StudyForrest					
V1d	-0.08	[-0.11, -0.05]	-5.57	**<.001**	1.60
V2d	-0.01	[-0.04, 0.02]	-0.85	.410	0.28
V3d	-0.04	[-0.05, -0.02]	-4.96	**<.001**	0.80
V1v	-0.05	[-0.07, -0.03]	-4.70	**<.001**	1.13
V2v	-0.07	[-0.09, -0.05]	-8.31	**<.001**	1.79
V3v	-0.01	[-0.02, 0.00]	-1.63	.252	0.24
Game of Thrones					
V1d	-0.03	[-0.04, -0.02]	-5.35	**<.001**	0.34
V2d	-0.05	[-0.06, -0.04]	-10.08	**<.001**	0.58
V3d	-0.06	[-0.07, -0.05]	-11.22	**<.001**	0.81
V1v	-0.00	[-0.01, 0.01]	-0.73	.469	0.04
V2v	-0.06	[-0.07, -0.05]	-16.28	**<.001**	0.99
V3v	-0.04	[-0.05, -0.03]	-9.43	**<.001**	0.63
Human Connectome Project					
V1d	-0.01	[-0.01 -0.01]	-7.21	**<.001**	0.11
V2d	-0.01	[-0.02 -0.01]	-9.42	**<.001**	0.14
V3d	-0.02	[-0.03 -0.02]	-15.64	**<.001**	0.26
V1v	-0.04	[-0.04 -0.04]	-24.67	**<.001**	0.44
V2v	-0.03	[-0.03 -0.03]	-18.73	**<.001**	0.31
V3v	-0.03	[-0.04 -0.03]	-19.77	**<.001**	0.37

5. Between-subject interhemispheric non-corresponding vs. intrahemispheric

As for within-subject comparisons, we also compared correlation magnitude between non-corresponding intrahemispheric regional pairings and intrahemispheric regional pairings in a between-subjects comparison. Comparisons were again conducted between the averaged non-corresponding interhemispheric correlations with the averaged intrahemispheric correlations, and the highest correlating non-corresponding interhemispheric regional pairing with the highest correlating intrahemispheric regional pairing.

Between-subject non-corresponding interhemispheric correlations were generated by correlating time-courses of activity for individual non-corresponding regions from two subjects' different hemispheres. (e.g., lOFA from P1:rFFA from P2). As before, This was repeated and averaged all possible subject pairings for each individual subject. Between-subject intrahemispheric correlations were generated in the same manner as described previously. These comparisons were repeated across all three datasets. Descriptive statistics are reported in Table 5.0, while inferential statistics are displayed in Sections 5.1 – 5.3 below.

Table 5.0

Between-subjects paired samples comparisons of non-corresponding interhemispheric average, interhemispheric highest, intrahemispheric average, and intrahemispheric highest means and (standard deviations) of Fishers' z correlations for three datasets.

Network & Region	Dataset & Connectivity Correlations											
	StudyForrest				Game of Thrones				H.C. Project			
	Inter Avg.	Inter High.	Intra Avg.	Intra High.	Inter Avg.	Inter High.	Intra Avg.	Intra High.	Inter Avg.	Inter High.	Intra Avg.	Intra High.
Face Selective Regions												
OFA	0.13 (0.02)	0.13 (0.02)	0.20 (0.04)	0.22 (0.03)	0.18 (0.04)	0.18 (0.04)	0.28 (0.08)	0.30 (0.06)	0.26 (0.05)	0.26 (0.05)	0.41 (0.07)	0.45 (0.08)
FFA	0.12 (0.02)	0.13 (0.02)	0.20 (0.04)	0.22 (0.03)	0.16 (0.04)	0.16 (0.03)	0.23 (0.05)	0.25 (0.05)	0.27 (0.05)	0.27 (0.05)	0.41 (0.07)	0.45 (0.08)

STS	0.10	0.11	0.17	0.19	0.16	0.17	0.28	0.30	0.19	0.20	0.28	0.30
	(0.02)	(0.02)	(0.04)	(0.05)	(0.04)	(0.04)	(0.08)	(0.06)	(0.04)	(0.04)	(0.06)	(0.06)
AMG	0.05	0.05	0.06	0.06	0.06	0.05	0.07	0.08	0.12	0.13	0.15	0.16
	(0.01)	(0.01)	(0.01)	(0.01)	(0.02)	(0.02)	(0.03)	(0.03)	(0.03)	(0.03)	(0.04)	(0.04)
Scene Selective Regions												
OPA	0.22	0.22	0.32	0.33	0.18	0.18	0.24	0.26	0.30	0.30	0.41	0.42
	(0.04)	(0.04)	(0.05)	(0.04)	(0.05)	(0.05)	(0.06)	(0.06)	(0.06)	(0.06)	(0.07)	(0.07)
PPA	0.21	0.22	0.32	0.33	0.21	0.22	0.24	0.26	0.33	0.34	0.41	0.42
	(0.04)	(0.04)	(0.05)	(0.04)	(0.05)	(0.05)	(0.06)	(0.06)	(0.06)	(0.06)	(0.07)	(0.07)
RSC	0.15	0.15	0.19	0.19	0.17	0.17	0.21	0.23	0.23	0.23	0.28	0.29
	(0.03)	(0.03)	(0.04)	(0.04)	(0.05)	(0.05)	(0.05)	(0.06)	(0.05)	(0.05)	(0.06)	(0.06)
Early Visual Regions												
V1d	0.21	0.23	0.27	0.31	0.30	0.31	0.35	0.36	0.39	0.40	0.44	0.46
	(0.03)	(0.03)	(0.05)	(0.05)	(0.06)	(0.07)	(0.08)	(0.08)	(0.08)	(0.09)	(0.09)	(0.10)
V2d	0.20	0.22	0.27	0.31	0.27	0.29	0.32	0.34	0.38	0.39	0.44	0.46
	(0.04)	(0.03)	(0.05)	(0.05)	(0.06)	(0.06)	(0.08)	(0.09)	(0.08)	(0.08)	(0.09)	(0.10)
V3d	0.23	0.24	0.26	0.30	0.26	0.27	0.29	0.32	0.39	0.40	0.42	0.43
	(0.04)	(0.04)	(0.06)	(0.04)	(0.06)	(0.06)	(0.06)	(0.08)	(0.08)	(0.08)	(0.09)	(0.09)
V1v	0.23	0.24	0.28	0.32	0.30	0.32	0.35	0.36	0.39	0.40	0.41	0.45
	(0.03)	(0.03)	(0.04)	(0.05)	(0.06)	(0.06)	(0.08)	(0.08)	(0.08)	(0.09)	(0.09)	(0.09)
V2v	0.22	0.23	0.30	0.34	0.27	0.28	0.31	0.33	0.38	0.40	0.42	0.46
	(0.02)	(0.03)	(0.04)	(0.04)	(0.06)	(0.06)	(0.07)	(0.07)	(0.08)	(0.08)	(0.09)	(0.10)
V3v	0.24	0.26	0.30	0.34	0.27	0.29	0.31	0.34	0.38	0.39	0.42	0.46
	(0.03)	(0.03)	(0.04)	(0.04)	(0.06)	(0.06)	(0.07)	(0.07)	(0.08)	(0.08)	(0.09)	(0.10)

5.1 Face Network

Across all regions, averaged non-corresponding interhemispheric correlations were commonly lower or indifferentiable from averaged intrahemispheric correlations. Of the higher magnitude intrahemispheric correlations, only the FFA reached significance in the StudyForrest dataset [$t(14) = -3.65$, $p = .021$, $d_{avg} = 0.37$]; in the Game of thrones dataset, both the OFA and STS reached significance (p ≤ .024); and in the Human Connectome Project dataset, the STS and AMG reached significance (p < .001). All other regions were indifferentiable between interhemispheric and intrahemispheric averages in correlation magnitude (p ≥ .142).

For the highest correlating regional pairings, non-corresponding interhemispheric correlations showed a consistent direction of being lower than intrahemispheric connectivity. This was significant (*p* < .001) across multiple regions, primarily in the Human Connectome Project dataset, but failed to reach significance for the OFA, FFA, and STS in the StudyForrest dataset (*p* ≥ .072), and the OFA, FFA and AMG in the Game of Thrones dataset (all *p* ≥ .050).

Table 5.1

Between-subjects paired samples comparisons of non-corresponding interhemispheric average, interhemispheric highest, intrahemispheric average, and intrahemispheric highest normalized time course of activity correlations (Zr) across face network ROIs for three datasets.

Network & Region	M_{diff}	95% CI	*t*	*p*	d_{avg}
**NC Interhemispheric Avg. vs. Intrahemispheric Avg.**					
StudyForrest					
OFA	-0.00	[-0.01, 0.00]	-1.30	.645	0.16
FFA	-0.01	[-0.01, -0.00]	-3.65	**.021**	0.37
STS	-0.01	[-0.01, -0.00]	-2.15	.196	0.32
AMG	-0.00	[-0.01, 0.00]	-1.29	.645	0.22
Game of Thrones					
OFA	-0.01	[-0.01, -0.00]	-3.04	**.024**	0.14
FFA	0.00	[-0.00, 0.00]	0.33	>.999	0.02
STS	-0.01	[-0.01, -0.00]	-3.36	**.014**	0.15
AMG	0.00	[-0.00, 0.00]	0.25	>.999	0.02

Human Connectome Project					
OFA	0.00	[-0.00, 0.00]	1.79	0.142	0.04
FFA	-0.00	[-0.00, 0.00]	-1.82	0.142	0.03
STS	-0.01	[-0.01, -0.01]	-8.69	**<.001**	0.21
AMG	-0.00	[-0.01, -0.00]	-6.3	**<.001**	0.16
**NC Interhemispheric Highest vs. Intrahemispheric Highest**					
StudyForrest					
OFA	-0.02	[-0.03, -0.00]	-2.88	.072	0.53
FFA	-0.02	[-0.03, -0.,00]	-2.88	.072	0.53
STS	-0.02	[-0.04, , 0.00]	-1.21	.645	0.37
AMG	-0.01	[-0.01, -0.00]	-4.61	**<.001**	0.55
Game of Thrones					
OFA	-0.02	[-0.04, -0.00]	-2.38	.088	0.31
FFA	-0.02	[-0.03, -0.01]	-4.66	**<.001**	0.34
STS	-0.02	[-0.04, -0.00]	-2.38	.088	0.31
AMG	-0.01	[-0.02, -0.00]	-2.7	.050	0.32
Human Connectome Project					
OFA	-0.04	[-0.05, -0.03]	-13.16	**<.001**	0.52
FFA	-0.04	[-0.05, -0.03]	-13.16	**<.001**	0.52
STS	-0.01	[-0.01, -0.01]	-6.18	**<.001**	0.22
AMG	-0.01	[-0.01, -0.01]	-12.3	**<.001**	0.32

5.2 Scene Network

For the OPA, averaged non-corresponding interhemispheric correlations were significantly lower than averaged intrahemispheric correlations in Human Connectome Project dataset [$t(174) = -5.85$, $p < .001$, $d_{avg} = 0.10$]; and Game of Thrones dataset [$t(44) = -3.47$, $p = .002$ $d_{avg} = 0.12$]; but failed to reach significance in the StudyForrest dataset [$t(14) = 0.39$, $p > .999$, $d_{avg} = 0.03$]. The PPA showed significantly greater intrahemispheric averaged correlations across all datasets (all $p < .001$). However, the RSC only showed no significant differences across any dataset (all $p \geq .100$).

For the highest correlating regional pairings intrahemispheric pairings also showed consistently higher magnitude correlations than interhemispheric pairings across the Game of Thrones and Human Connectome project datasets (all $p < .001$), but did not reach significance in this direction in the StudyForrest dataset (all $p \geq .244$).

Table 5.2

Between-subjects paired samples comparisons of non-corresponding interhemispheric average, interhemispheric highest, intrahemispheric average, and intrahemispheric highest normalized time course of activity correlations (Zr) across scene network ROIs for three datasets.

Network & Region	M_{diff}	95% CI	t	p	d_{avg}
**NC Interhemispheric Avg. vs. Intrahemispheric Avg.**					
StudyForrest					
OPA	0.00	[-0.01, 0.01]	0.39	>.999	0.03
PPA	-0.01	[-0.02, -0.01]	-5.55	**<.001**	0.31
RSC	-0.01	[-0.01, -0.00]	-2.62	.100	0.18
Game of Thrones					
OPA	-0.01	[-0.01, -0.00]	-3.47	**.002**	0.12
PPA	-0.01	[-0.01, -0.01]	-6.71	**<.001**	0.18
RSC	0.00	[-0.00, 0.01]	0.86	.395	0.03
Human Connectome Project					
OPA	-0.01	[-0.01, -0.00]	-5.85	**<.001**	0.10
PPA	-0.00	[-0.00, -0.00]	-6.98	**<.001**	0.06
RSC	-0.00	[-0.00, -0.00]	-0.35	.729	0.01
**NC Interhemispheric Highest vs. Intrahemispheric Highest**					

StudyForrest					
OPA	-0.01	[-0.02, 0.00]	-2.04	.244	0.27
PPA	-0.01	[-0.02, 0.00]	-2.04	.244	0.27
RSC	-0.00	[-0.01, 0.01]	-0.53	>.999	0.05
Game of Thrones					
OPA	-0.02	[-0.02, -0.01]	-8.58	**<.001**	0.32
PPA	-0.02	[-0.02, -0.01]	-8.58	**<.001**	0.32
RSC	-0.02	[-0.02, -0.01]	-9.01	**<.001**	0.34
Human Connectome Project					
OPA	-0.01	[-0.01, -0.01]	-6.08	**<.001**	0.10
PPA	-0.01	[-0.01, -0.01]	-6.08	**<.001**	0.10
RSC	-0.01	[-0.01, -0.01]	-12.37	**<.001**	0.16

5.3 Early Visual Network

Across all regions, averaged non-corresponding interhemispheric correlations were significantly lower than averaged intrahemispheric correlations (all $p \leq .024$).

For the highest correlating regional pairings, this direction remained consistent, with a significantly greater magnitude intrahemispheric correlations throughout the majority of regions (all $p \leq .016$), except for the paired V1d and V1v in the Game of Thrones dataset, which failed to reach significance [$t(44) = -1.88$, $p = .134$, $d_{avg} = 0.12$].

Table 5.4

Between-subjects paired samples comparisons of non-corresponding interhemispheric average, interhemispheric highest, intrahemispheric average, and intrahemispheric highest normalized time course of activity correlations (Zr) across early visual network ROIs for three datasets.

Network & Region	M_{diff}	95% CI	t	p	d_{avg}
**NC Interhemispheric Avg. vs. Intrahemispheric Avg.**					
StudyForrest					
V1d	-0.01	[-0.02, -0.01]	-7.24	**<.001**	0.44
V2d	-0.01	[-0.02, -0.00]	-2.89	**.024**	0.42
V3d	-0.01	[-0.02, -0.01]	-5.11	**<.001**	0.34
V1v	-0.01	[-0.02, -0.00]	-3.46	**.016**	0.35
V2v	-0.01	[-0.02, -0.00]	-2.84	**.024**	0.33
V3v	-0.01	[-0.02, -0.01]	-4.20	**.007**	0.51
Game of Thrones					
V1d	-0.01	[-0.02, -0.01]	-8.37	**<.001**	0.21
V2d	-0.01	[-0.01, -0.01]	-7.59	**<.001**	0.21
V3d	-0.01	[-0.01, -0.01]	-3.82	**<.001**	0.15
V1v	-0.01	[-0.02, -0.01]	-9.45	**<.001**	0.23
V2v	-0.01	[-0.02, -0.01]	-9.17	**<.001**	0.25
V3v	-0.02	[-0.02, -0.01]	-12.96	**<.001**	0.32
Human Connectome Project					
V1d	-0.01	[-0.01, -0.01]	-18.07	**<.001**	0.14
V2d	-0.01	[-0.01, -0.01]	-8.05	**<.001**	0.08
V3d	-0.01	[-0.01, -0.01]	-10.71	**<.001**	0.09
V1v	-0.02	[-0.02, -0.02]	-22.58	**<.001**	0.2
V2v	-0.01	[-0.01, -0.01]	-16.53	**<.001**	0.17
V3v	-0.01	[-0.02, -0.01]	-16.57	**<.001**	0.18
**NC Interhemispheric Highest vs. Intrahemispheric Highest**					
StudyForrest					
V1d	-0.04	[-0.05, -0.02]	-4.37	**.007**	0.72
V2d	-0.04	[-0.05, -0.02]	-4.37	**.007**	0.72
V3d	-0.04	[-0.06, -0.01]	-3.38	**.016**	0.72

V1v	-0.03	[-0.04, -0.02]	-6.77	**<.001**	0.78
V2v	-0.04	[-0.05, -0.03]	-8.23	**<.001**	1.04
V3v	-0.04	[-0.05, -0.03]	-8.23	**<.001**	1.04
Game of Thrones					
V1d	-0.01	[-0.02, 0.00]	-1.88	.134	0.12
V2d	-0.02	[-0.03, -0.01]	-3.28	**.006**	0.24
V3d	-0.03	[-0.03, -0.02]	-6.07	**<.001**	0.37
V1v	-0.01	[-0.02, 0.00]	-1.88	.134	0.12
V2v	-0.02	[-0.03, -0.01]	-4.73	**<.001**	0.28
V3v	-0.02	[-0.03, -0.01]	-3.81	**<.001**	0.34
Human Connectome Project					
V1d	-0.02	[-0.02, -0.01]	-12.4	**<.001**	0.16
V2d	-0.02	[-0.02, -0.01]	-12.4	**<.001**	0.16
V3d	-0.01	[-0.02, -0.01]	-10.18	**<.001**	0.16
V1v	-0.03	[-0.04, -0.03]	-19.07	**<.001**	0.37
V2v	-0.04	[-0.04, -0.04]	-23.6	**<.001**	0.42
V3v	-0.04	[-0.04, -0.04]	-23.6	**<.001**	0.42

6. Within-subject vs between-subject analysis

Idiosyncrasy of corresponding interhemispheric and intrahemispheric correlations was calculated via by performing a paired-sample *t*-test on the within-subjects vs. the between-subjects differences (*Corresponding Interhemispheric correlation* (Zr) – *Highest Intrahemispheric pairing correlation* (Zr)).

Across all face and scene regions, the within-subject interhemispheric correlations differences were significantly greater than the between-subject interhemispheric correlation differences (all $p \leq .046$).

Early Visual regions showed a reverse of this pattern, with a direction towards lower within-subject interhemispheric correlation differences. This was significant for no regions in the StudyForrest Dataset (all $p > .174$); but this difference was significant across the Game of Thrones and Human Connectome Project datasets (all $p \leq .018$) with two exceptions. These exceptions were from V1d and V2d in the Human Connectome project dataset. While V2d did not reach significance in the typical direction [$t(173) = -1.19$, $p = .235$, $d_{avg} = 0.13$] , V1d showed a unique reversal of the pattern found in other early visual regions, showing greater within-subject interhemispheric correlation differences [$t(173) = 4.76$, $p < .001$, $d_{avg} = 0.50$].

Table 6

Idiosyncrasy testing for ROIs across three studies, calculated as regional residual (interhemispheric – highest intrahemispheric) connectivity, compared within- and between-subjects.

	StudyForrest			Game of Thrones			Human Connectome P.		
Region	*t*(14)	*p*	d_{avg}	*t*(44)	*p*	d_{avg}	*t*(173)	*p*	d_{avg}
Face network									
OFA	2.41	**.046**	0.81	2.78	**.008**	0.55	7.46	**<.001**	0.70
FFA	5.63	**<.001**	2.00	4.07	**<.001**	0.83	5.93	**<.001**	0.54
STS	4.72	**<.001**	1.44	3.72	**.002**	0.74	14.96	**<.001**	1.32
AMG	2.54	**.046**	0.94	9.42	**<.001**	1.99	18.09	**<.001**	1.77

Scene Network									
OPA	7.23	**<.001**	2.45	10.01	**<.001**	2.01	36.55	**<.001**	3.80
PPA	9.08	**<.001**	3.18	8.92	**<.001**	1.81	13.93	**<.001**	1.36
RSC	10.3	**<.001**	3.75	17.11	**<.001**	3.57	59.66	**<.001**	6.13
Early Visual Network									
V1d	0.06	.952	0.02	-2.73	**.018**	0.54	4.76	**<.001**	0.50
V2d	-2.42	.174	0.81	-2.64	**.018**	0.55	-1.19	.235	0.13
V3d	-1.62	.384	0.55	-6.71	**<.001**	1.38	-6.18	**<.001**	0.67
V1v	-1.02	.648	0.33	-3.28	**.006**	0.71	-4.85	**<.001**	0.5
V2v	-1.80	.372	0.69	-5.84	**<.001**	1.2	-10.26	**<.001**	1.05
V3v	-2.22	.215	0.79	-6.76	**<.001**	1.41	-26.11	**<.001**	2.60
